# Spatial and Topological Organization of DNA Chains Induced by Gene Co-localization

**DOI:** 10.1371/journal.pcbi.1000678

**Published:** 2010-02-12

**Authors:** Ivan Junier, Olivier Martin, François Képès

**Affiliations:** 1 Epigenomics Project, Genopole, CNRS UPS 3201, UniverSud Paris, University of Evry, Genopole Campus 1 - Genavenir 6, Evry, France; 2 Institut des Systèmes Complexes Paris Île-de-France, Paris, France; 3 Université Paris-Sud, UMR 8626 LPTMS, F-91405, Orsay, France; 4 Université Paris-Sud, UMR 0320/UMR 8120 Génétique Végétale, Gif/Yvette, France; The Hebrew University, Israel

## Abstract

Transcriptional activity has been shown to relate to the organization of chromosomes in the eukaryotic nucleus and in the bacterial nucleoid. In particular, highly transcribed genes, RNA polymerases and transcription factors gather into discrete spatial foci called transcription factories. However, the mechanisms underlying the formation of these foci and the resulting topological order of the chromosome remain to be elucidated. Here we consider a thermodynamic framework based on a worm-like chain model of chromosomes where sparse designated sites along the DNA are able to interact whenever they are spatially close by. This is motivated by recurrent evidence that there exist physical interactions between genes that operate together. Three important results come out of this simple framework. First, the resulting formation of transcription foci can be viewed as a micro-phase separation of the interacting sites from the rest of the DNA. In this respect, a thermodynamic analysis suggests transcription factors to be appropriate candidates for mediating the physical interactions between genes. Next, numerical simulations of the polymer reveal a rich variety of phases that are associated with different topological orderings, each providing a way to increase the local concentrations of the interacting sites. Finally, the numerical results show that both one-dimensional clustering and periodic location of the binding sites along the DNA, which have been observed in several organisms, make the spatial co-localization of multiple families of genes particularly efficient.

## Author Summary

The good operation of cells relies on a coordination between chromosome structure and genetic regulation which is yet to be understood. This can be seen in particular from the transcription machinery: in some eukaryotes and bacteria, transcription of highly active genes occurs within discrete foci called transcription factories, where RNA polymerases, transcription factors and their target genes co-localize. The mechanisms underlying the formation of these foci and the resulting topological structure of the chromosome remain to be elucidated. Here, we propose a thermodynamic framework based on a polymer description of DNA in which genes effectively interact through attractive forces in physical space. The formation of transcription foci then corresponds to a self-organizing process whereby the interacting genes and the non-interacting DNA form two phases that tend to separate. Numerical simulations of the model unveil a rich zoology of the topological ordering of DNA around the foci and show that regularities in the positions of the interacting genes make the spatial co-localization of multiple families of genes particularly efficient. Experimental testing of the predictions of our model should shed new light on the relation between transcriptional regulation and cellular conformations of chromosomes.

## Introduction

The proper genome-wide coordination of gene expression has been shown to be linked to the spatial organization of genes within the cell [1],[2]. This can be seen in particular from the transcription machinery: in some eukaryotes [3],[4] and bacteria [5], transcription of highly active genes occurs within discrete foci called transcription factories, where RNA polymerases, transcription factors (TFs) and their target genes co-localize. In eukaryotes, genes that are co-localized in the same nuclear area are thought to participate to the same developmental function [2]. Accordingly, one-dimensionally distant genes, *i.e.* genes that are far apart along the DNA, participating in the same cellular function are expected to co-localize in the three-dimensional cellular space during periods of active transcription, as has been shown for generally active genes [6],[7].

It has been argued that the associated higher concentrations of certain molecular species allow for more efficient transcription regulation [8], just as having transcriptional factories allows for more rapid recycling of the molecular components of the RNA polymerase complex; both of these aspects justify a posteriori conformational organizations of the DNA to produce co-localization phenomena. On the experimental side, the 3-dimensional architecture of eukaryotic [1],[9],[10] and prokaryotic [11]–[13] chromosomes has been under active study. Yet, the fine structure at the level of the transcription factories and the role of chromosome architecture in the regulation of transcription remain to be elucidated. Several of the important open questions are: (1) What is the mechanism that localizes genes at their transcription factories? (2) What is the corresponding topology of the 3-dimensional chromosomal structure? (3) Have gene positions along DNA been selected during evolution so that they can be more easily co-localized in space during transcription? In this article, we propose a general framework to address these questions.

Let us first recall the two main scenarios that have been proposed for the topological organization of chromosomes and transcription factories. In the *solenoid* framework [14], the chromosome forms a ring, torus or solenoid, visiting the different foci periodically. The foci result from the bridging of distant binding sites *via* the binding of bivalent transcription factors. In support of this scenario, genes regulated by the same TFs in yeast and *Escherichia coli*, plus genes belonging to phylogenetically conserved gene pairs in *E. coli* have been shown to arise with some periodicity along the DNA [15]–[17]. A solenoidal pattern would then generate higher local concentrations of the TF and of its DNA binding sites, and thus might allow more efficient transcriptional regulation [14]; if so, this should translate into a selective advantage by analogy to the *lac* operon case [8]. In another framework, hereafter referred to as the *rosette* scenario [18], the DNA chain first forms loops around one single transcription factory; then, a succession of such rosettes might form a necklace of rosettes. Interactions between DNA bound proteins, and depletion forces due to the presence of large complexes (the transcription factories) that are surrounded by numerous small entities (from water molecules to proteins), have been proposed to be responsible for the formation of DNA loops [19]. From a regulatory point of view, the 3-dimensional structure of DNA has been proposed to modulate the transcription process according to the position of the genes within the loops [20].

In both scenarios, TFs are expected to play a crucial role since, just as in the *lac* operon case, bivalent TFs can bridge distant sites; a multimerized form of TFs can also facilitate the bridging [21],[22]; the interaction with the transcription factories, and more generally with active RNA polymerases [23], is also expected to induce the bridging. In fact this may be so even if the binding sites are very distant when measured along the one-dimensional DNA, in direct analogy with the numerous examples of the stabilization of DNA loops via the binding of bivalent [8],[24],[25] or multimerized [21],[22],[26] TFs. From a theoretical point of view, a stabilizing interaction between distant binding sites can lead to the emergence of large agglomerates of bridged sites [27]. Unfortunately, neither computational nor theoretical work has addressed the consequences of such bridging forces on the spatial organization of these agglomerates, and hence, on the resulting chromosomal organization.

In this work, we investigate the folding properties of a single self-avoiding polymer chain along which specific sites interact according to a short range potential, thus mimicking an attraction mediated by either transcription factors or large protein complexes. In such a context, we show that transcription factories can be viewed as the result of self-organization, the process consisting of a micro-phase separation between interacting and non-interacting sites. Using Monte-Carlo simulations, we show that a rich variety of topologies are likely to describe the spatial co-localization of genes. Moreover, our results strongly suggest that if genes are to co-localize into families according to their function or regulatory control, a regular pattern of gene positions along the DNA is necessary.

All the parameters that are necessary for understanding both the modeling framework and the subsequent biological implications are listed in Table 1. A short explanatory note is provided for each parameter.

**Table 1:** List of parameters.

Parameter	Name and description
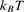	*Thermal energy:* energy unit reflecting the thermal agitation of the environment (*e.g.* the nucleoplasm) in which the polymer (DNA or chromatin) resides.
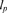	*Persistence length:* distance beyond which the polymer loses most of its orientational order.
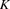	*Bending modulus:* energy per unit length. It reflects the energetic cost to locally bend the polymer, which leads to a persistence length 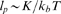 .
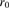	*Hard-core radius:* radius of the hard-core polymer. Electrostatic repulsions between DNA segments are therefore modeled as simple hard-core repulsions.
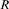	*Gyration radius:* spatial extension of a spherical globule conformation of the WLC. 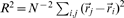 where 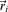 is the position of the 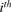 monomer.
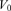	*Binding free energy:* typical free energy gain due to the bridging of two distal sites along the DNA, not taking account the entropy change of the distant parts of the chain.
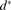	*Interaction range:* Distance below which two interacting sites interact. In such case, they lower the energy of the system by an amount of 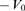 ( 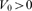 ).
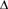	Mean distance between two successive interacting sites along the DNA.
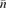	Maximum number of partners of a single interacting site. Biologically speaking, this can be viewed as the maximum number of TF binding sites of a regulated gene.
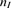	Typical number of genes that are transcribed simultaneously in a transcription factory (biological data). This corresponds, here, to the mean number of interacting sites belonging to the discrete foci.
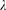	Mean distance between two consecutive sites. For one type of interacting sites, 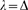 .

### Model

Statistical properties of long DNA chains in good solvents are accurately described by worm-like chain (WLC) models [28]. These types of models provide a coarse-grained description of protein-coated DNA (*e.g.* the eukaryotic chromatin). They are simple enough to allow some analytical treatment and to be investigated numerically. They include the typical elastic behavior of DNA, which has been measured *in vitro* and *in vitro*.

**Figure 1:**
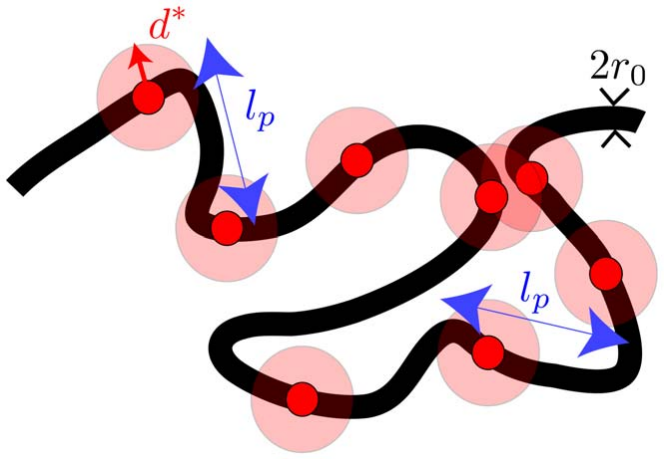
2D cartoon of the three-dimensional self-avoiding WLC model with sparse interacting sites. Sites that can interact are represented by small red filled circle. The outer red circles define the interaction range 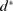 of the potential. The persistence length 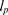 (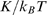) is the typical length beyond which the polymer loses most of its orientational order. See Figure S1 for further details on the polymer description.

More precisely, the WLC model is defined by a bending energy 
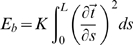
 where 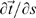 is the variation of the tangent vector along the curvilinear abscissa 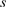 of the polymer, 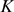 is the bending modulus, and 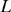 is the total length of the polymer. In our study, we further take into account the short range electrostatic repulsion of DNA (DNA is negatively charged). Due to the screening of the charges *in vivo*, it is commonplace to model this repulsion as a hard-core potential. Our framework therefore consists of a self-avoiding WLC with a hard-core radius 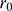. The persistence length 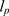 along the polymer is defined as the distance beyond which the WLC loses most of its orientational order – see Fig. 1. For an infinitely thin chain (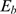 is then the only energy), one has 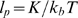 where 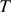 is the temperature in Kelvin and 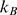 is the Boltzmann constant. For naked DNA, 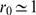 nm; moreover typical *in vivo* ionic conditions lead to 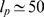 nm [29], though 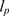 may appear larger or smaller due to the presence of DNA bound proteins such as histone like proteins. In the case of eukaryotes, one can model the 30 nm chromatin fiber by taking 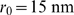; 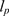 can vary between 50 and 250 nm, depending on the compaction level of the chromatin.

Within this framework, genes along the DNA are associated with specific sites on the polymer (Fig. 1). Part of these genes will participate to the co-localization process. In this regard, several possible scenarios have been proposed (see the introduction). Here, we investigate the effect of thermodynamic interactions (*e.g.* van der Walls or ionic) between proteins and DNA and discuss whether these interactions can lead to a well coordinated self-organization of the chromosome. We therefore do not consider proteinic complex assemblings that require energy consumption nor possible active forces – *e.g.* induced by molecular motors – that would drive chromosome loci to the transcription factories. Finally, the binding of proteins on DNA is treated implicitly, that is, two chromosome loci that can be bridged by a proteinic complex interact according to a short-range attractive potential 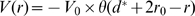. Here 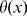 is the step function that is 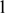 if 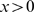 and 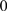 otherwise, 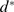 is the interaction range, and 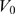 is the strength of the potential. This interaction mimics a free energy term resulting from the bridging of the chromosome, either by bivalent TFs such as the Lac repressor [8], or by TF multimerization such as in the 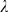 phage [26]; similarly, tethering may be mediated by the transcription factories, or more generally by RNA polymerase/TF complexes, which occur for instance during the transcriptional activation of some bacterial genes [30]; values of 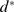 therefore lie between several nanometers and several tens of nanometers. The free energy gain comprises that due to protein-DNA binding and, when multimerization comes into play, that due to protein complex formation. In any case, free energies (*i.e.*, 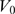) are expected to be a few kcal/mol (and thus a few 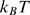) [25],[26].

Our coarse-graining procedure allows to tackle, within the same formalism, different mechanisms that may lead a gene to be an interacting site. For instance, our model can mimic the effect of the chromatin condensation (heterochromatin), which can prevent a site from participating to the interaction just by hiding it or making it unaccessible. A more realistic modeling of chromosome structuration would include heterogeneities in the interaction between the sites (different 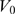 for different pairs of sites) and also the explicit presence of solvent molecules. However, our goal here is to provide a plausible general picture for the formation of transcription factories that can be cast within a formalism as simple as possible.

Overall, our framework consists of a self-avoiding WLC along which specific sites are distributed sparsely and are able to interact (Fig. 1). In this context, we define 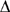 as the mean distance between two successive interacting sites along the DNA. We also define the capacity of a site as the number of other sites it can interact with simultaneously. For the results shown here, we take for simplicity no limit on the capacity. The maximum number of partners of a site, hereafter referred to as 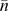, will be limited only by the steric constraint: one cannot pack more than some maximum number of sites within a given distance of a point. Notice that the possibility of multiple interactions is compatible with the fact that gene regulatory regions frequently have several TF binding sites. Moreover, large protein complexes, which are likely to appear around transcription factories, should favor the simultaneous interaction of several binding sites.

Our results can be divided into three parts. First, we show that transcription factories can be viewed as the result of a micro-structuration mechanism, which is an archetype of a self-organizing process. In particular, our calculation highlights the range of parameters for which the micro-structuration is expected. Next, we use numerical simulations to address the topological ordering of DNA around the transcription factories. Finally, we tackle the problem of forming transcription factories in the presence of different families of interacting sites, *i.e.* families corresponding to different regulatory properties.

## Results

### Transcription factories as a micro-phase separation

Before dealing with the mechanisms that are responsible for the formation of discrete foci, we quickly recall the basic phenomenology of a self-attracting and self-avoiding WLC. Within the framework of our model, this corresponds to considering a dense distribution of interacting sites along the DNA, so the interacting sites are close-by along the whole DNA. Such WLCs have been extensively studied for more than forty years [31],[32]. Depending on the values of the parameters, they mainly lead to three typical conformations, which are also known to arise for chromosomes *in vitro* and *in vivo* [33],[34]. First, in the absence of the self-attracting interaction, the WLC behaves as a self-avoiding random walk, at least on length scales larger than the persistence length; this leads to the so-called “swollen” state – in the physics of an isolated polymer chain, some phases may arise only when the polymer is short; as suggested in [35], we refer to these pseudo-phases as “states”. Second, introduce an attractive interaction. For a sufficiently strong attraction, the polymer goes to one of two possible compact conformations. The densest conformation is obtained by having the polymer wind many times around a circle, forming in effect a kind of torus; accordingly, this has been called the “toroidal” state [32]. For a weaker interaction, less dense and less ordered conformations arise, the so-called “globules” where the polymer forms a ball but otherwise seems rather random. Which of these macroscopic states – swollen, toroidal or globule – describes the equilibrium state depends on the parameters, the two most important ones being the attractive force and the polymer stiffness [35].

**Figure 2:**
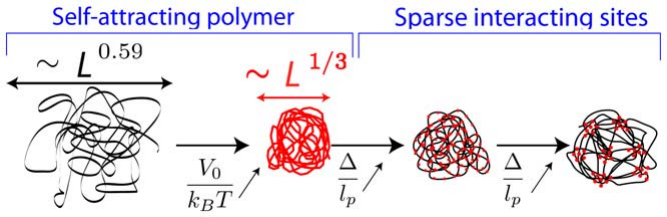
Sketch of the micro-phase transition in a single polymer chain. Active interacting sites are indicated by red points. From left to right: Starting from a self-attracting WLC in the swollen state, a sufficient increase of 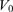, which is indicated by the upward arrow, can lead to the formation of a (compact) homogeneous self-attracting globule. Starting from the latter with a sufficiently high value of 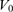, a progressive increase of the distance 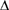 between the interacting sites along the polymer (upward arrows) will lead to less compact globules, and eventually to the formation of a micro-structured globule with co-localized sites.

Coming back to our system consisting of a single chain with sparse interacting sites, its peculiarity is that only *a few designated sites* of the chain are subject to the attraction; this means that a further organization of the chain on smaller length scales can arise as now explained. Suppose that the interacting sites are sparsely distributed along the polymer. Starting in one of the compact states, the energy can be enhanced by *local* rearrangements, keeping the polymer compactness roughly unchanged. At a coarse-grained level, one can focus on the local density of the interacting sites in three-dimensional space. In a random conformation, the density will be uniform. By contrast, after local rearrangements, the density will vary, leading to clumping in some areas, and voids in others. In essence, a uniform density is energetically unstable, and so the system will spontaneously structure so as to form regions of high and low densities of interacting sites (Fig. 2). This leads to a micro-phase separation between interacting and non-interacting sites, which is reminiscent of what is observed in block co-polymers [32].

In the following, we investigate in detail this micro-structuration, tackling the problem in two ways. First, we use a mean-field theory of polymer physics. This allows us to qualitatively capture the transition between the homogeneous states with a uniform distribution of interacting sites and the micro-structured states with a spatially modulated distribution of interacting sites. Next, we use Monte-Carlo simulations to both validate our analytical results and to further study the DNA conformations around the foci.

### State diagram in biologically relevant situations

**Figure 3:**
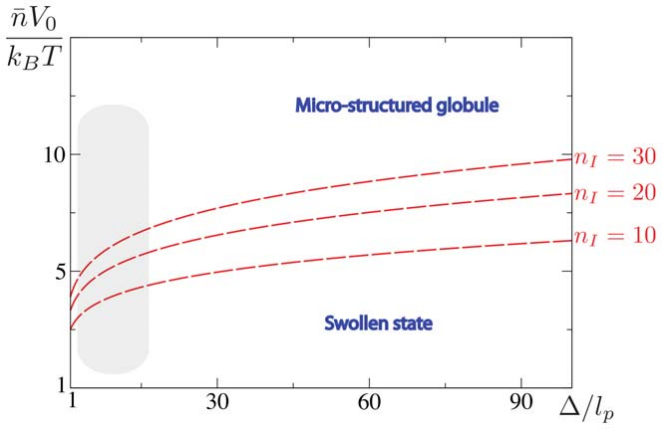
Macroscopic phase diagram in biologically relevant conditions. Spatial co-localization of co-regulated genes as modeled by a flexible WLC composed of sparse interacting sites, that is having 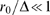 and 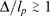. In the case where the attractive interaction of the WLC is not too strong, the macroscopic state diagram of the system contains two states (leaving apart the 3-dimensional organization of the foci): the micro-structured globule and the swollen state. Fixing 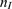, the number of sites that belong to the discrete foci in the micro-structured state, the transition lines (dashed red curves) separating the swollen state from the micro-structured globule are of the form 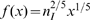 – see relation (3). The gray area indicates the typical values taken by 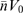 and 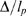 in the eukaryote case. Notice that given an estimation of 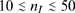 [37], different biologically relevant values of 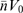 can allow switching from one state to the other one.

Within the scope of chromosome structuration *via* the bridging of co-regulated genes, the macroscopic state diagram for not too strong attractive forces is limited to two states: the swollen state and the micro-structured globule – see Fig. 3. Generally speaking, the micro-structured globule tends to be favored thermodynamically over the homogeneous globule for interacting sites that are sparsely distributed along the WLC; the homogeneous density of binding sites is unstable to a modulation, at least if the capacity of sites is not too small. In this situation, the number of interacting sites lying within the foci of the micro-structured globule (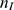), which is kept fixed in our calculation for the sake of simplicity, determines the position of the transition between the two states. We now present the principles of the underlying calculation, emphasizing the crucial parameters that determine the balance between the states. This allows us to discuss the mechanisms responsible for the shift between the states.

### Strategy

The best way to determine the thermodynamically favored state is to compute the free energy of each state as a function of the model parameters, which is explicitly done in section 3 in Text S1; the state with the lowest free energy is the favored one. In the following, for the sake of simplicity, we do not tackle the issue of the toroidal phase, considering only the micro-structured globule, the swollen state and the homogeneous globule. Ignoring their internal structure, these isotropic states look like balls. As a consequence, they can be characterized by a radius 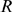 and a free energy 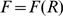. In the most general case, the free energy can be decomposed into four terms:
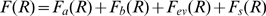
(1)

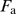
 is the free energy due to the attractive potential between the interacting sites. 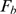 is the contribution from the bending energy. 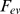 is the free energy cost due to the excluded volume of the polymer within an area of extension 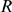, which stems from the repulsion of the hard-core monomers constituting the polymer. 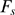 is the entropy related to the number of polymer configurations that are compatible with a radius 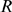 [32].

For a given type of organization (*e.g.* a micro-structured globule), the free energy calculation consists in first determining the 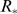 that minimizes the free energy. Then, 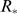 is plugged into the free energy relation Eq. (1), which gives the corresponding free energy of the state, that is 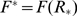. One must compare the free energies 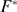 of each state. The explicit dependence on the radius 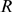 of each term is calculated using a standard mean-field single chain polymer theory, also known as Flory theory [32], focusing on the bulk contribution to the free energies (large 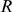). In the following, we skip the technical details and give the final results of the calculation, as well as its interpretation. For more details on the derivation, we refer the reader to section 3 in Text S1.

In the case of a sparse distribution of interacting sites, the position of the sites may be crucial, as we shall see in the last section. To simplify our discussion, we therefore consider, in a first stage, sites that are regularly spaced by 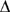 along the DNA.

#### Homogeneous states

The calculation shows that the balance between the homogeneous globule and the swollen states rests on the value of the single parameter (see sections 3.3 and 3.4 in Text S1):
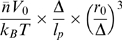
(2)Accordingly, three mechanism can be responsible for the greater stability of one state compared to the other. First, there is the competition between the attractive potential coming from the interacting sites on the one hand, and the destabilizing thermal energy coming from the solvent on the other. This corresponds to the term 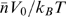; notice that the effective free energy of attraction per site is proportional to the maximum number of partners of a site (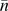). Second, 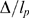 reflects the difficulty for rigid polymers (large 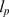) to bridge interacting sites that are close by along the polymer. Finally, the ability of the polymer to form contacts between interacting sites crucially depends on the number of these sites. This is reflected by the term 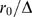, which corresponds to the linear density of the interacting sites along the polymer.

Thus, the swollen state is more stable whenever the above parameter is small compared to 1, that is, at high temperature, for rigid polymers, and when few interacting sites are present along the polymer. In the opposite case, *i.e.*, at sufficiently low temperatures, for sufficiently flexible polymers, and for a sufficient number of interacting sites, the homogeneous globule becomes more stable.

#### The micro-structured globule

As we shall see below, in biological situations in which transcriptionally regulated genes are involved, the swollen state is always more stable than the homogeneous globule. In these conditions, the micro-structured globule is the most stable state if and only if it is more stable than the swollen state. To tackle this point, for the sake of simplicity, we suppose: i) that the foci are composed of 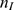 interacting sites where 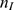 is uniform across the whole globule, and ii) that two nearest-neighbor foci are separated by a distance that is also uniform across the whole globule. These hypotheses are mean-field-like since they neglect spatial variations of certain characteristics of the polymer. For 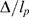 not too small, which is appropriate for gene co-localization (see below), one can show that stability depends on the value of the single parameter (see section 3.5 in Text S1):
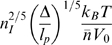
(3)For low (respectively high) values of this parameter, *i.e.*, when 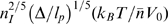 is much smaller (respectively much larger) than 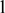, the micro-structured globule is more (respectively less) stable.

Three ingredients are therefore crucial for the stability of the micro-structured globule. First, big foci (large 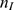
's) tend to be less stable than small foci, although a rigorous calculation would require 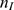 not to be fixed *a priori*: nothing prevents foci from splitting if this lowers their free energy. Notice that the number of sites per foci is expected to be limited from above by the hard-core properties of the polymer (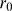), and by the properties of the interaction as well (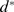). This can be checked by numerical simulations.

Next, some amount of rigidity seems to be necessary in order to stabilize the micro-structured globule since small values of 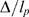 tend to lower the value of the above parameter. This may appear counter-intuitive with respect to what has been stated in the previous section, namely, that rigid polymers tend to favor swollen states. This last statement is true but the results presented in this section are valid only when 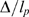 is not too small, *i.e.*, when 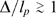. In this limit, which is the one of biological relevance to our problem, the more rigid the polymer, the lower the excluded volume coming from the non-attracting parts of the polymer. Indeed, little space is available for the polymer to fluctuate in between the foci. Hence, a rigid polymer would tend to diminish the fluctuations so that the hard-core repulsions between the monomers would diminish (with an increase of the distance between the foci). Overall, this would tend to stabilize the micro-structured globule. Nevertheless, very large values of the rigidity, *i.e.* 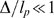, would eventually destabilize the micro-structured globule to give way to the swollen state. In any case, due to the low value of the exponent 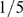 in relation (3), the effect of varying 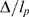 on the state diagram is rather modest, at least in the biological situations we are interested in – see Fig. 3. Finally, the above parameter shows that strong attracting interactions (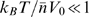) naturally tend to favor the micro-structured globule.

### Application: gene co-localization and transcriptional regulation

Our WLC offers a single framework to discuss the formation of transcription factories both in bacteria and in eukaryotes. Within the context of transcriptional regulation, genes participating to the same transcription factories are believed to participate to specific cellular functions. 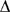 can therefore be evaluated as the typical distance between two consecutive genes that are co-regulated by the same TF or that are known to participate to the same function. As a consequence, 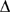 is expected to be larger than the distance separating two genes, *e.g.* 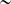
1 kbps in bacteria (*i.e.*, 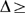
300 nm), and 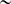
100 kbps in mammals (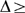
600 nm) – we have used 150 bps/nm for the chromatin fiber [36]. This leads to factors 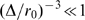 in relation (2). Hence, within the scope of our model, for biologically relevant values of 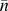 and 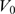, the homogeneous globule state (with a uniform distribution of actively transcribed genes) is thermodynamically unlikely both in bacteria and eukaryotes.

As far as the micro-structured globule with discrete foci is concerned, in eukaryotes one can approximate 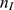 as the typical number of active RNA polymerases within one transcription factory, *i.e.*, 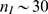 [37]. By considering 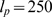 nm and 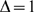 Mbps (6 mm), which corresponds to the mean distance between two consecutive genes regulated by a TF in the human genome [38], one finds 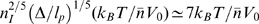. The regulatory regions of eukaryotic genes often have several TF binding sites of the same type, which can be interpreted as 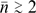. Hence, a bridging induced by TFs (with binding energies of several 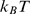
's per TF), or induced by a proteinic complex involving TFs, is sufficient to induce the formation of transcription factories according to the above micro-phase separation (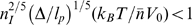). Moreover, given the parameters of chromatin, the values of 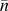 and 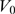 lie in a range that allows to switch between a state with discrete foci and the swollen state – see Fig. 3. This suggests that the micro-phase structuration is also a possible mechanism for fine tuning the global genetic regulation of a cell.

In bacteria, an interesting case concerns the formation of the putative transcription factories during the transcription of rRNA operons [5]. In this situation, 7 operons scattered along 2 Mbps have to be co-localized. 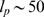 nm then leads to 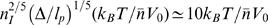. In the same way, both co-regulated genes [15] and genes that are thought to be functionally related [17] have been shown to be periodically spaced according to a 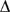 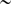
100 kbps period. Supposing these genes are co-localized by groups of at least ten, this leads again to 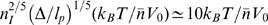. Hence, in bacteria, if one considers one single binding site per gene, large binding energies are required for the formation of transcription factories. However, this should be balanced by the overall negative supercoiling of bacterial DNA which is beyond the scope of our model. Together with the action of nucleoid-associated proteins (*e.g.* histone like proteins such as Fis, H-NS or HU), this effect would tend to condense the chromosome and hence to dampen consequences of thermal fluctuations.

### DNA organization of the micro-structured globule

**Figure 4:**
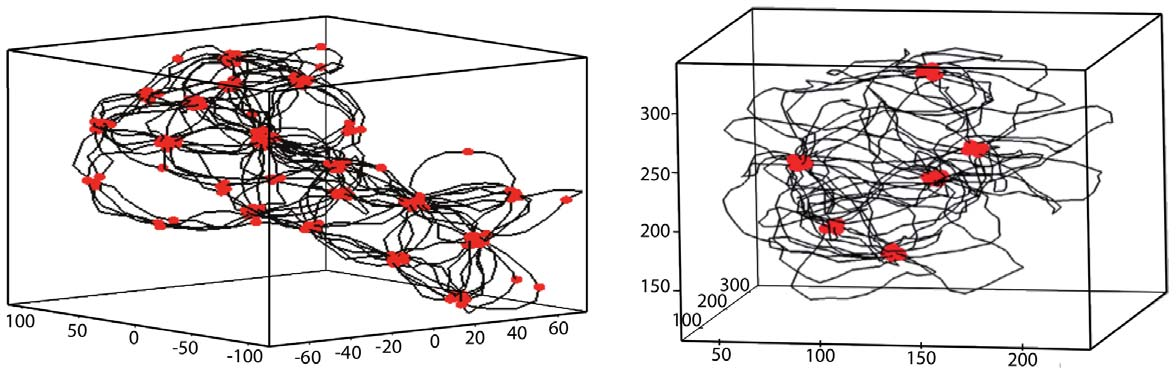
States with micro-structuration of interacting sites. Parameters correspond to the case of naked DNA with 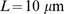 and 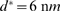. Left panel : 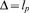, 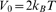. Right panel: 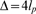, 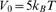. Foci are typically composed of 10 interacting sites. Axes give positions in nanometers.

Numerical simulations of polymer models are useful to investigate the principles of chromosome organization within space [19],[39],[40]. In this respect, simulations of our self-avoiding WLC (see Methods for details) confirm that gene foci arise for persistence lengths, binding free energies and inter-gene distances that are typical of bacteria and eukaryotes (see Fig. 4 for two such examples). Simulations are also useful to see how the foci organize in 3-dimensional space. Indeed, *a priori*, foci may form regular lattices, random lattices, or they may wander with time. In this respect, our results suggest a rich variety of equilibrium conformations that depend on the parameters of the system. However, from a computational point of view, we are not able to investigate the *thermodynamic* state diagram when the DNA chain becomes relatively large because the different metastable states last the whole time window of the simulation once they are formed; in particular, we do not see switches between the states as would arise in a situation of co-existence. Thus we are limited to considering the most likely structures that form when starting from a random coil (swollen) configuration as we progressively increase the value 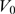 from an initial zero value. Note that from a biological point of view, such metastable states may be just as relevant as the true equilibrium states.

The resulting structures can be divided into three main groups, as we now describe.

#### The micro-structured solenoids

**Figure 5:**
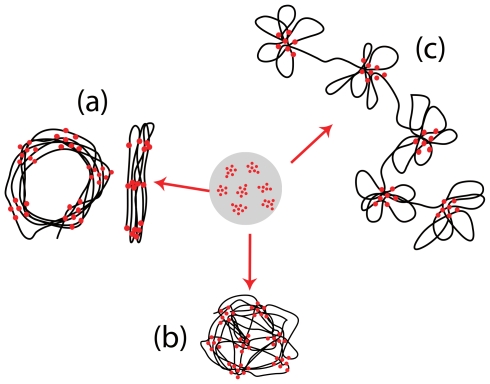
Naive expectation of finite size conformations. When distances (along the one-dimensional DNA) between interacting sites are large enough, discrete foci can form in space. This cartoon shows different possible organizations of the foci and of the DNA chains. (a) Foci and bundles of DNA free of interacting sites are organized along one (thick) dimension. They form either solenoidal structures or open linear structures. (b) Foci belong to nodes of a spatial network of DNA bundles free of interacting sites. In this situation, the DNA chain goes from one focus to another focus that is in its spatial vicinity. (c) Foci are organized along one dimensional necklace while DNA chains form rosette structures.

**Figure 6:**
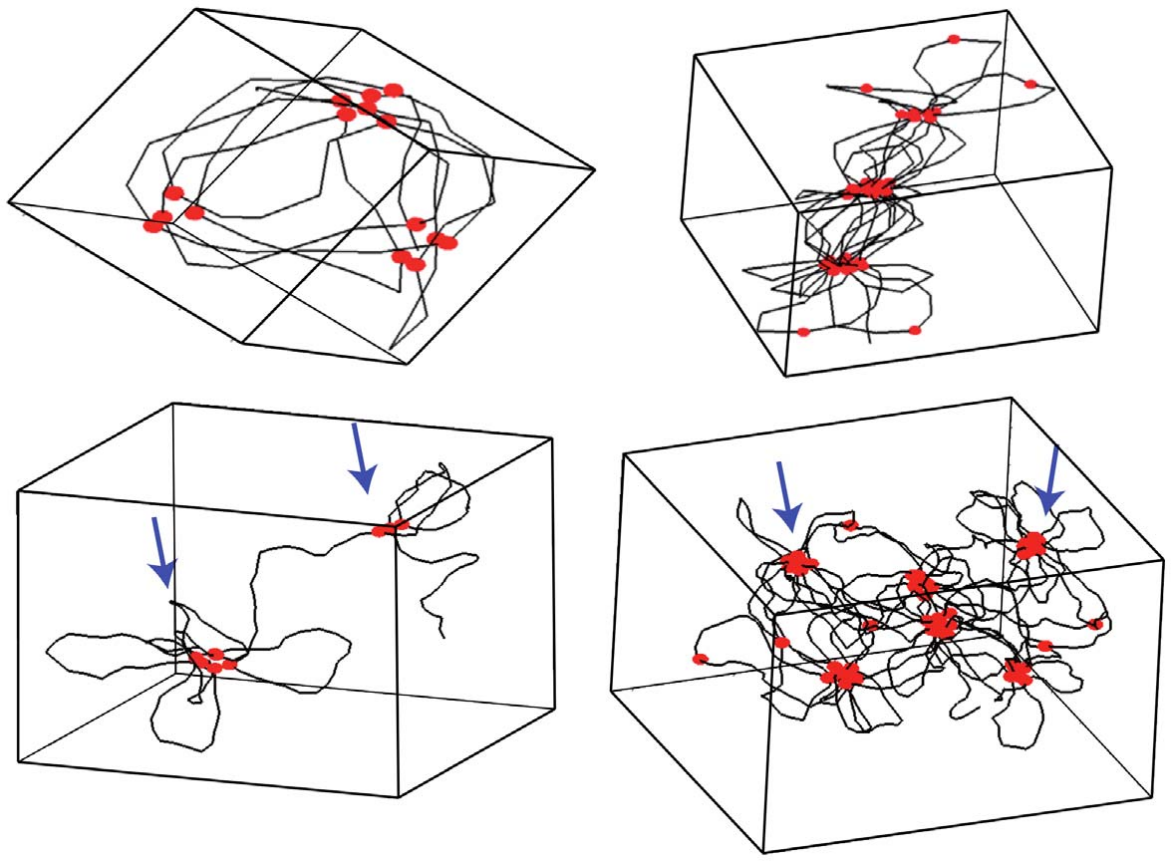
Topological ordering of DNA around the foci. *Upper panels:* Conformations topologically equivalent to solenoids where foci and DNA bundles are organized in a one-dimensional manner. Naked DNA parameters: 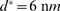, 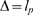 and 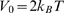. Left panel: 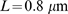; right panel: 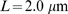. *Lower panels:* Rosettes. Nodes are considered as rosettes (blue arrows) when more than half of their outgoing DNA chains come back to the same node at the next interacting site. Chromatin fiber with 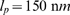, 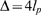. Left panel: for small sizes (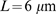), necklaces of no more than two rosettes appear. Right panel: for larger sizes (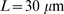), foci tend to form random spatial networks instead of long necklaces of rosettes.

Begin with a toroidal conformation of a self-attracting WLC and try to maximize the number of sites in interaction when 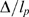 increases. To do that, one can take the sites and push/slide them so that they co-localize in sections of the torus, *i.e.*, agglomerate in foci along planes that cut the torus through its small section (Fig. 5a). One can obtain ring-like structures or open linear structures that are topologically equivalent. To differentiate these structures from the uniform toroidal conformations, we refer to them as *solenoidal*. This type of organization has been advocated by Képès in 2003 to justify the tendency for genes that are co-regulated by the same TFs to be periodically positioned along the DNA [14], and by Wright *et al.* in 2007 to explain periodic trends in the position of phylogenetically conserved gene pairs in bacteria [17]. In the limit of extremely compact DNA, the number of planes is determined by the periodicity parameter 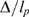 and by the size of the torus. As can be seen in Fig. 6 from the numerical simulation of the WLC, such rings of gene foci and topologically equivalent open conformations arise for some parameters of the WLC that are relevant for describing naked DNA.

#### The rosette structure

If 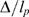 is increased, one can reduce the number of foci by putting more binding sites in each. At some point, there will remain a single focus if it has the capacity to hold all the binding sites. In this situation, the polymer performs round trips about one single focus as shown in Figure S2. In the case of several foci, it may be that successive interacting sites on the polymer belong to the same focus before going on to another one (Fig. 5c). This is the kind of structure advocated by Cook et al. [18] for DNA organization around transcription factories, the loops of DNA being tethered to one focus. We shall call it a “necklace of rosettes” because each focus corresponds to a rosette. Do such structures arise in our simulations of the WLC? For most of the parameter values we studied, we have never observed more than 2 successive rosettes (see Fig. 6 for such a situation when parameters are set to correspond to the eukaryotic case). However, more rosettes may arise when several types of interaction are present, as we shall see in the next section.

#### The traveling chain structure

The previous solenoidal and rosette structures have a significant entropic cost; if energetic effects cannot compensate this, one expects these two structures to be destabilized. As reported in Figs. 4 and 6, our numerical results show that for long polymers, bundles of chain segments free of interacting sites may form a spatial network while the interacting sites are concentrated within the nodes (see Video S1 showing the three-dimensional realization). Within this network, when going from one binding site to the next one along the DNA chain, one typically moves to a different focus. Accordingly, we call this structure “the traveling chain” structure.

#### Topological state diagram

**Figure 7:**
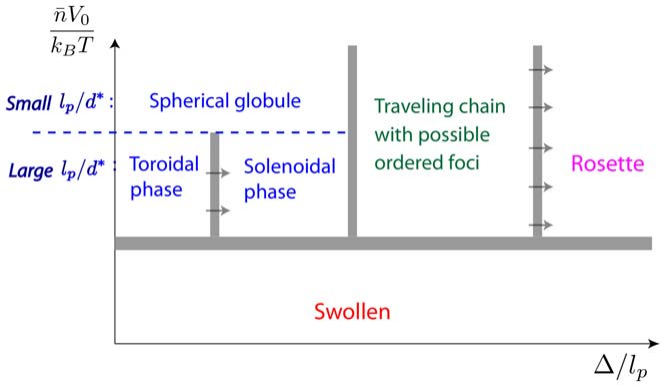
Qualitative state diagram for finite length WLC. Computational tools can provide qualitative insights of the state diagram as a function of the system parameters. In this regard, the thick gray lines in the diagram point out the expected transitions as the parameters are varied – they do not provide the precise form of the transition lines. The horizontal blue dashed line is used to simultaneously discuss two limiting cases: very flexible polymers (small 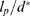) and very rigid polymers (large 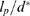). As far as self-attracting WLC are concerned, at low temperature, the former tend to form spherical globules, whereas the latter tend to form toroids [35]. Now, working with a fixed chain length, our results show that for large enough values of 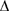, the rosette is the most stable state thermodynamically. Starting from a single rosette, a further decreasing of 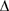 leads to the formation of several foci. These foci can be organized according to an isotropic spatial network (*i.e.*, the traveling chain conformation) as shown in Fig. 4, but also according to an anisotropic shape as shown in Figure S3. Thus, we go from rosettes to traveling chains when 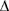 decreases, whatever the values of the rigidity. In this respect, the transition lines separating the rosette and the multi-foci conformations are expected to lower as the length of the polymer increases (arrows in the figure). In the same way, as indicated by arrows too, the solenoidal phase decreases in stability as the length of the polymer increases.

A schematic view of the resulting state diagram is depicted in Fig. 7. Interestingly, the three topological orderings (rosette, solenoid, traveling chain) can also be distinguished mathematically by a novel order parameter. Its construction is based on following the successive DNA binding sites which belong to various foci (see section 4.1 in Text S1 for the mathematical formulation); the successive steps can be thought of as a random walk, leading to 3 kinds of behaviors. For the necklace of rosettes, the walk visits the same focus multiple times but then goes away “for ever”: the random walk is “transient”. The other two cases correspond to “recurrent” random walks. In the case of the toroidal ordering, the walk does not visit the same focus twice in a row but comes back to the same focus after a (large) number of steps (the recurrence property). The traveling chain also gives a recurrent walk, but in contrast to the toroidal case, has a finite probability of revisiting the same focus within a few steps. Notice also that the interacting sites do not necessarily co-localize into spherical foci. Depending on the parameters, they can also organize themselves according to one-dimensional shapes (Figure S3).

Finally, recall that to simplify our study, we have used interacting sites that were periodically spaced along the DNA. Our results are robust to deviations from this case: small amounts of disorder do not change the possible states – see section 4.1 in Text S1. In the case of a fully random distribution of the gene positions, for small contour length 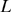 we observe rosette structures (with still one or two foci only) whereas large 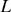 seems to favor the formation of spatial networks of foci.

### Generalization to multiple kinds of binding sites

Recent experiments in monkey Cos7 cells have shown that different transcription factories recruit different genes depending on their promoter type [41]. In the same spirit, one may hypothesize that genes regulated by the same TFs preferentially co-localize in space [6],[7],[14]. This would explain for instance, in yeast, the tendency of co-regulated genes to be clustered along the chromosomes [14],[42],[43]. A somewhat analogous issue arises in bacteria: one often finds that a TF coding gene, the binding site of that TF, and the corresponding regulated gene(s) are all close-by along the DNA (see [16] and references therein). This is thought to optimize the three-dimensional targeting process of the TF toward its binding site because, in bacteria, protein translation occurs close to the coding gene. Accordingly, space co-localization of distant binding sites for each TF type may very well occur since it is a natural way to make three-dimensional targeting and assembly of complexes more efficient. The investigation of gene positions in *E. coli* and yeast suggests that in these organisms a near periodic arrangement on the DNA of co-regulated genes may be at the base of a good 3-dimensional spatial co-localization [15],[16]. However, there are hundreds of TF types both in bacteria and yeast, and thousands in higher eukaryotes so that the satisfaction of all the separate co-localization constraints may be a hard problem for the organism to solve.

We have used our framework to numerically model the spatial co-localization process when 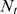 different types of TFs regulate a large number of genes. Specifically, we have 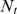 types of binding sites, where two binding sites interact only if they are of the same type. The way these sites (and their types) are positioned along the chain can affect the way the different foci form. We have therefore compared the co-localization process using four kinds of positioning of these binding sites, namely: i) sites ordered – and thus clustered – according to their types ii) randomly distributed sites and types; iii) periodically distributed sites and types; iv) sites that are spaced according to random multiples of 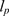, hereafter referred to as *random periodic*: there is approximate periodicity in the site positions while the site types are taken to be completely random (see Figure S4 for an illustrative explanation). Situation 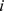 corresponds to the one-dimensional clustering of nearby binding sites whereas situations 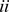 to 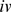 correspond to the interaction of binding sites that can be distant from each other. In particular, situation 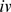 is useful to determine whether regularity in the site types is necessary for co-localization, even if there is some regularity in the site positions along the DNA. In this context, the mean distance 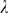 (measured along the chain) between two consecutive sites regardless of their type is a useful additional parameter to characterize the one-dimensional site properties along the DNA. To simplify our study, we take a number of interacting sites that is roughly the same for each site type so that 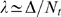, 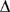 being the mean distance between two sites of the same type.

#### One-dimensional clustering *vs.* periodic spacing induced topologies

**Figure 8:**
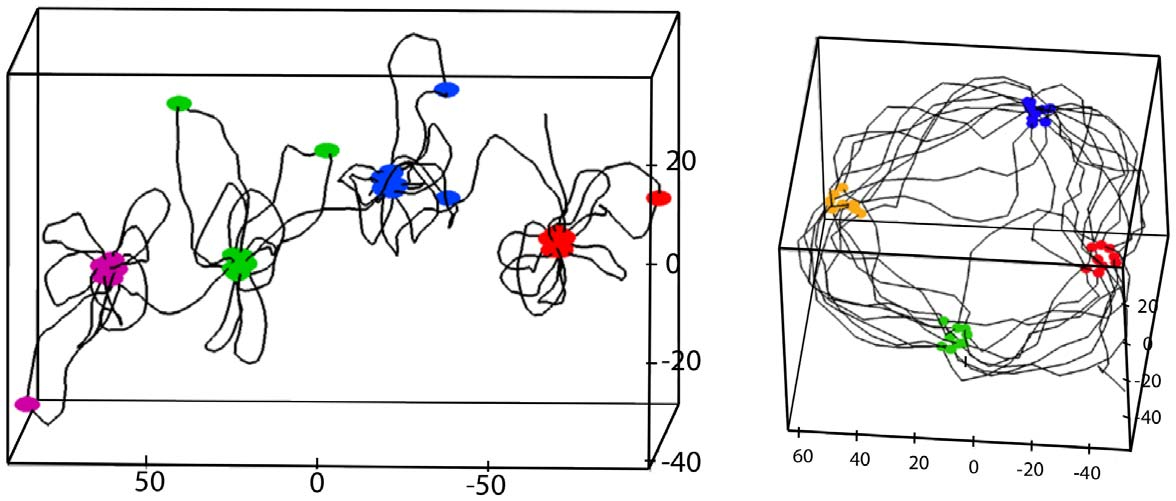
Impact of genome organization on chromosome structure. When several types (indicated by different colors of points) of binding sites are present, the specific positioning of the binding sites has a critical effect on the nature of the chromosomal structuring. For instance, binding sites that are ordered along the DNA according to their type, which can be viewed as a clustering of the binding sites along the DNA, favor the formation of rosettes. This is illustrated in the left panel (chromatin fiber, 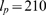 nm, 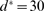 nm, 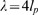, 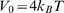, 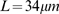). On the other hand, a periodic positioning tends to favor a solenoidal organization of the DNA as illustrated in the right panel (naked DNA. 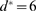 nm, 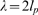, 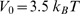, 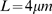).

The topological organization of DNA in response to the activation of transcription can dramatically depend on the organization of genes along the chromosome. Fig. 8 reports typical chromosome configurations, both for chromatin and naked DNA, that are obtained when the sites are ordered according to their types (case 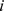) and when they are periodically spaced (case 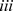). Both genomic organizations lead to a rapid formation of homogeneous transcription factories in space. Periodic spacing induces the formation of solenoidal configurations while one-dimensional clustering induces the formation of necklaces of rosettes.

#### Periodic site positions favor co-localization of distant sites

Our results show that some periodicity in the site positions allows for an efficient spatial co-localization of distant sites and also that a too disordered positioning hinders the formation of foci. As shall be explained now, this can be seen in both the dynamic and static aspects of the folding transition of our polymer model.

**Figure 9:**
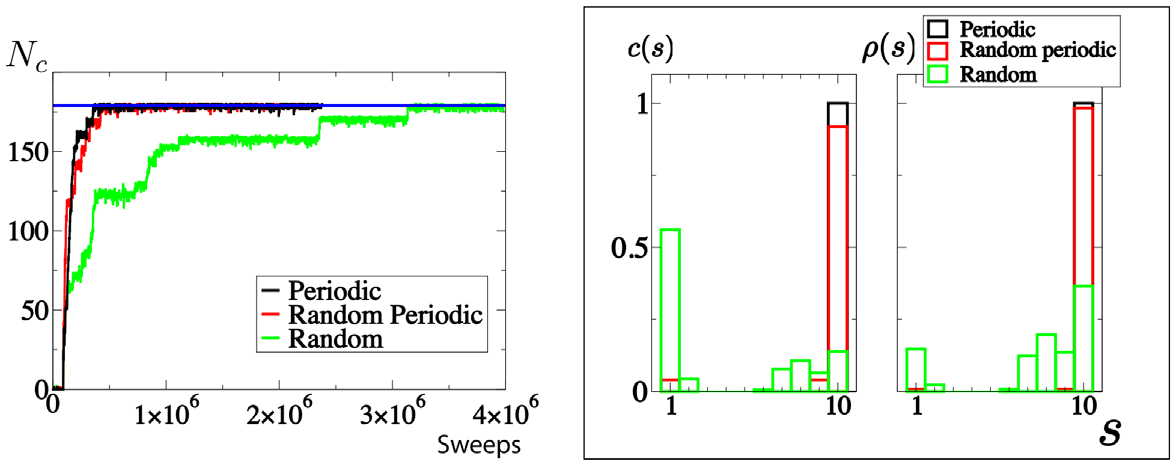
Impact of genome organization on the formation of transcription factories. *Left panel:* Folding trajectories traced by the temporal evolution of the total number 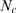 of contacts that are established among interacting sites. The time is counted in number of sweeps (cf. Methods). The plateau gives the maximum number of contacts. Naked DNA. 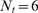, 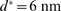, 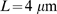 and 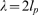. *Right panel:* Comparison of the steady state cluster composition between periodic, random periodic and random site positioning. 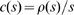 is the cluster size distribution. 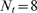, 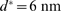, 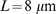 and 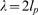. See text for the definition of 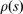.

First of all, the folding transition from an unstructured state to a steady state takes a longer time in the random case than in the periodic cases. This is illustrated in Fig. 9 using single trajectories. In general, the larger 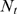, the larger this effect. Moreover, as expected, the folding times tend to become similar when 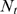 decreases at fixed 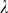 or when 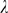 increases at fixed 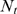.

**Figure 10:**
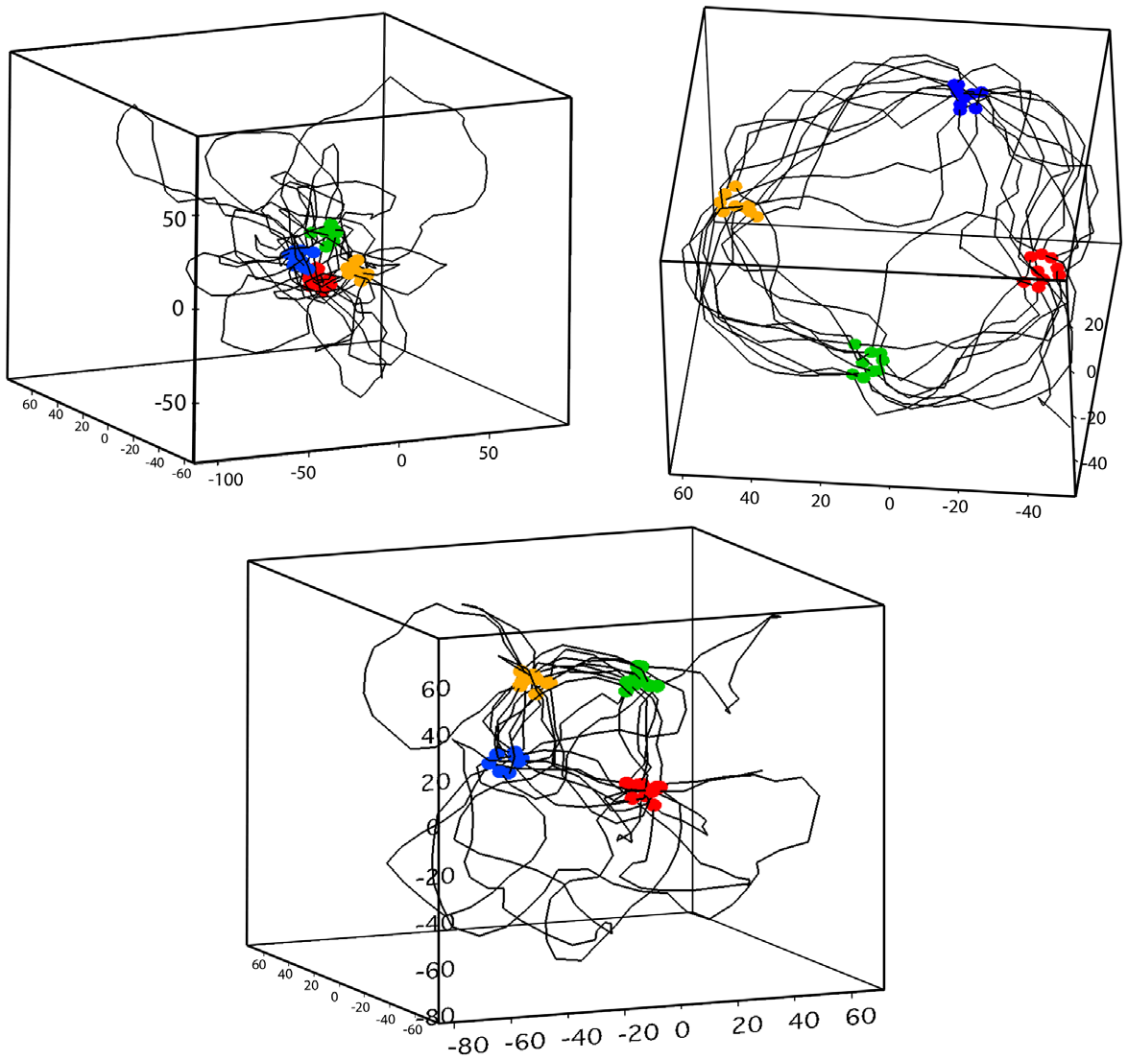
Snapshot of DNA conformations and foci within steady states. Here, foci of maximum sizes are reached in all cases. The global conformation depends on the way the sites were laid out on the chain. *Upper left panel*: random positions; *Upper right panel*: periodic positions; *Lower panel*: periodic random positions. Naked DNA. 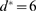 nm. 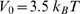. 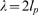. 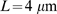.

Second, the fraction 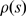 of binding sites belonging to a focus of size 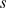 in a steady state depends on the organization of the interacting sites along the DNA (Fig. 9). For pure periodic positions, the polymer forms either a solenoidal structure or a well organized network of foci for long chains which succeed in clustering all the binding sites. In this situation, 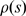 has a single peak at large sizes 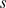. This pure periodic case can be viewed as an ideal context for forming specialized factories. Interestingly, our results further suggest that partial periodicity in the position of the sites (*i.e.*, case 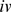 with an imperfect periodic organization) is sufficient to have an efficient spatial co-localization mechanism for which all foci have more or less the same size, *i.e.*, 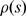 exhibits mainly a single peak as shown in Fig. 9. In contrast to the pure periodic case, the spatial structure is not a ring-like structure although there is some circularity in the structure (Fig. 10). When positions are drawn randomly (case 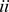), 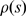 becomes bimodal for large values of 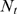 and fixed values of either 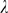 or 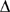. In particular, a peak at 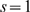 appears, the other main peak corresponding to large values of 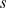. Hence, a finite fraction of the sites remains isolated in space, *i.e.*, many sites do not belong to a so-called transcription factory, even though large clusters are formed (Figure S5). Moreover, by running different trajectories from different random initial configurations, we have observed that in the random case the steady states differ from run to run, *i.e.*, the way the sites cluster varies, although the parameters of the polymer model are kept fixed (data not shown). Overall, the situation is reminiscent of a thermodynamic glass transition where the equilibrium free energy is dominated by multiple thermodynamic states that are separated by high energy barriers. In such situations, frustration, which is due here to the presence of binding sites along the polymer that are incompatible with a ring-like structure, is a crucial feature for constraining the thermodynamic state.

Third and lastly, it is interesting to compare the spatial conformations of the structured state for the random and the periodic positioning of the interacting sites. As can be seen from Fig. 10, if positions are randomly drawn, the clusters tend to form close to each other in space whereas in the pure periodic case, the clusters are well separated and are periodically spaced along a torus. Moreover, in the random periodic case, the clusters are also well separated although no specific ring structure is formed. Overall, these results show that some regularity in the positions of distant interacting sites is needed to have well separated foci in space, which presumably is a pre-requisite for a good operation of transcription factories.

## Discussion

Within a fairly general framework, we investigated the topological organization of a model chromosome. Using an effective attractive potential between selected genes on a DNA chain, we found that these could organize into discrete foci, with the DNA visiting the foci in several topologically distinguishable ways. The foci are composed of genes that can be far away from each other along the DNA, which is supported by the recent observation of numerous Mbps-range DNA loops [6],[7].

Of course, *in vivo*, numerous obstacles might prevent chromosomes from achieving the conformations we predict: supercoiling, chromatin remodeling and confinement introduce other interactions that may dominate for some parameter values. Another point is that we have focused on equilibrium conformations whereas in reality cellular processes operate away from equilibrium. However, a pure equilibrium approach is useful because it shows the natural organizational trend of the system.

Several conclusions transpire from our framework. First, in bacteria and eukaryotes, the formation of transcription factories may be related to a self-organizing process akin to the folding transition of single polymer chains. The underlying thermodynamic mechanism is a spatial micro-phase separation driven by regions of DNA where genes are subject to similar transcriptional regulation. In effect, due to the very nature of the self-avoiding DNA chain, all genes cannot cluster together to enhance transcription rates; instead, discrete foci must form in space. Our results therefore confirm that self-organization may play a crucial role in the structuring of chromosomes [10],[44].

Interestingly, the interaction strength needed between distant sites along the DNA in order to induce the micro-structuration is compatible with the binding of TFs to DNA. The bridging can be achieved *via* a bivalent TF, or more generally through the formation of large protein complexes, *e.g.* by tethering the DNA-bound TF to ongoing transcription factories. This corroborates TFs as possible entities for mediating the effective attractive potential; our model therefore predicts a 3D co-localization of co-regulated genes. In eukaryotes, this can be tested by a combination of 3D fluorescence in situ hybridizations (FISH) and chromosome conformation capture techniques [45] as exemplified in [6],[7],[45],[46]. In bacteria, this can be tested by using the site-specific recombination system of the bacteriophage 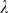 [13]. Furthermore, as illustrated by Eq. (3), the number of co-regulated genes that can be co-localized within the same focus depends both on the number of TF binding sites per gene and on the binding energies. This leads to the prediction that the presence of aptamers which can compete with TFs for binding to cognate DNA sites will lead to smaller transcription factories or even none at all.

Second, using numerical simulations of our model, we have shown that the topology of the DNA conformations fall into several classes according to the way the foci are visited, and that two of these classes had been previously hypothesized on the basis of biological evidence. For instance, starting from a toroidal organization of DNA which has been observed in some organisms [34], if the interacting sites that stabilize this structure become less dense, there should be a micro-phase separation whereby distinct foci appear along the ring, which fits the solenoidal model proposed in [14]. As interacting site density decreases further, rosettes may form as proposed in [18]. Or the DNA may successively visit the different foci in a random fashion, corresponding to our “traveling chain” topology.

Third, which topological ordering arises generally depends on the way the binding sites are positioned along the one-dimensional DNA. We find that some periodic regularities and some clustering in the positioning of co-regulated genes, as observed respectively in [15],[16] and in *e.g.* [42],[43], strongly favor the formation of well-separated foci with a homogeneous size and content, and disfavor the presence of genes outside of the foci. To this end, we considered the possibility of having multiple types of protein binding sites, thought to be associated with different transcription factor families or gene functions. We found that having periodically-positioned targets of multiple TFs favored the solenoidal topology whereas the necklace of rosettes topology was favored if groups of genes were one-dimensionally clustered along the DNA (Fig. 8).

## Methods

### Numerical implementation of the WLC model

Numerical simulations of the continuous self-avoiding WLC model are based on an off-lattice semi-flexible polymer composed of 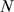 jointed cylinders of radius 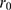 and length 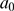 (Figure S1). The cylinders are impenetrable (hard-core interactions) and two consecutive cylinders 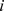, 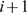 that form a bending angle 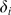 contribute a bending energy 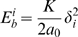 to the total energy 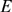. The solvent is implicit, it is not treated explicitly.

Interacting sites are taken to be located at the joints between two consecutive cylinders; a joint can contain or not an interacting site. They interact *via* a uniform short range square potential of depth 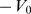 and interaction range 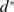 (Figure S1). Thus, if two non-consecutive interacting sites 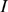 and 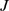 can interact, they contribute an energy 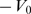 if the distance 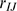 between them is less than 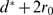. As a result, the total energy of the system reads:
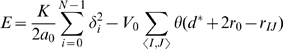
(4)where 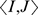 means that the non-consecutive interacting sites 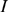 and 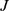 are able to interact. 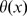 is the step function that is equal to 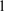 if 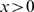 and is 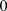 otherwise. 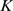 is the bending modulus of the polymer; it depends on the type of polymer (DNA or chromatin) that is described. To have results that are insensitive to the discrete nature of the polymer representation, one should use a segment length that is a small fraction of the persistence length; in all our simulations, we take this factor to be one fifth. Note also that it is best to work *off-lattice* as lattice anisotropy is known to induce geometrical artifacts that could spoil the interpretation of the results.

The persistence length and the radius of our polymer representation of DNA depend on the type of organism to be modeled. In the limit of an infinitely thin polymer (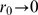), the persistence length 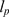 is related to the bending modulus via 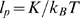. In the case of the self-avoiding polymers presented in this work, this relation holds well (data not shown) so that the bending energy is enough to define 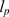.

### Monte Carlo simulation

To sample the state space of our polymer model, we use standard Monte Carlo procedures with the Metropolis accept/rejection rule, which guarantees reaching thermodynamic equilibrium if ergodicity is not broken. The Monte Carlo method consists in 1) picking at random two joints (a joint being the point where two consecutive cylinders coincide), and 2) applying a 3-dimensional rotation around the axis that passes through the two joints according to a random angle in 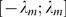. Here we take a relatively small value, 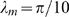 (at larger values the acceptance rate goes down).

### Polymer time scales and steady states

The largest timescale for the conformational relaxation of a single coiled polymer scales as 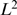 [31]. From a numerical point of view, this results in a relaxation times that scales as 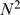 for local microscopic evolution rules, *i.e.*, where only a finite number of cylinders are updated at each time step. In our Monte Carlo simulation, time is counted in number of sweeps, one sweep consisting of 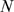 attempts to rotate part of the chain; also 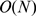 monomers are updated during one single rotation. This leads to a relaxation time that scales as 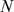. Nevertheless, we still need roughly 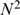 computer operations to thermalize the system in the regime of interest where interaction effects are dominant. This prevents the current method from scaling up to very long chromosomes, although we can deal with interesting systems. We present simulations with up to 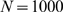 cylinders; this corresponds to 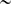
50 kbps in the case of naked DNA and 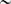
5 Mbps in the case of the chromatin fiber. In this case, we are not able to sample the equilibrium space of the condensed polymer because the different metastable states are very stable.

In situations of slow temporal evolution, defining a steady state may be a tricky operation. For the parameters we used, our results suggest to consider as steady a state that lasts for more than 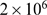 sweeps. For random positions of the interacting sites, the folding time can exceed 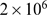 sweeps. Notice then that 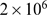 sweeps correspond to 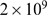 Monte-Carlo steps for 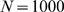 (the largest size we report here).

## Supporting Information

Text S1 4 sections 1. Physics of self-avoiding worm like chains 2. Framework and calculation strategy 3. Free energy calculations 4. Insights into the condensed phases
(0.70 MB PDF)

Figure S1 Discrete cylinder approximation of a self-avoiding worm-like chain model
(0.09 MB PDF)

Figure S2 Rosette conformations
(0.10 MB PDF)

Figure S3 Anisotropic structuring of the interacting sites
(0.14 MB PDF)

Figure S4 Choices for positioning the interacting site along the polymer
(0.05 MB PDF)

Figure S5 Typical spatial conformation for a random positioning of the sites
(0.12 MB PDF)

Video S1 Spatial organization of a traveling chain conformation - Naked DNA; d = 6nm; delta = 4; V0 = 5kT; L = 10um
(3.02 MB MOV)

## References

[1] Cremer T, Cremer M, Dietzel S, Muller S, Solovei I (2006). Chromosome territories – a functional nuclear landscape.. Curr Opin Cell Biol.

[2] Sexton T, Schober H, Fraser P, Gasser S (2007). Gene regulation through nuclear organization.. Nat Struct Mol Biol.

[3] Jackson DA, Hassan AB, Errington RJ, Cook PR (1993). Visualization of focal sites of transcription within human nuclei.. EMBO J.

[4] Wansink DG, Schul W, van der Kraan I, van Steensel B, van Driel R (1993). Fluorescent labeling of nascent RNA reveals transcription by RNA polymerase II in domains scattered throughout the nucleus.. J Cell Biol.

[5] Cabrera JE, Jin DJ (2003). The distribution of RNA polymerase in *Escherichia coli* is dynamic and sensitive to environmental cues.. Mol Microbiol.

[6] Osborne CS, Chakalova L, Brown KE, Carter D, Horton A (2004). Active genes dynamically colocalize to shared sites of ongoing transcription.. Nat Genet.

[7] Simonis M, Klous P, Splinter E, Moshkin Y, Willemsen R (2006). Nuclear organization of active and inactive chromatin domains uncovered by chromosome conformation capture-on-chip (4C).. Nat Genet.

[8] Müller-Hill B (1998). The function of auxiliary operators.. Mol Microbiol.

[9] Lanctôt C, Cheutin T, Cremer M, Cavalli G, Cremer T (2007). Dynamic genome architecture in the nuclear space: regulation of gene expression in three dimensions.. Nat Rev Genet.

[10] Misteli T (2005). Concepts in nuclear architecture.. Bioessays.

[11] Sherratt DJ (2003). Bacterial chromosome dynamics.. Science.

[12] Thanbichler M, Wang SC, Shapiro L (2005). The bacterial nucleoid: a highly organized and dynamic structure.. J Cell Biochem.

[13] Éspéli O, Boccard F (2006). Organization of the *Escherichia coli* chromosome into macrodomains and its possible functional implications.. J Struct Biol.

[14] Képès F, Vaillant C (2003). Transcription-based solenoidal model of chromosomes.. Complexus.

[15] Képès F (2003). Periodic epi-organization of the yeast genome revealed by the distribution of promoter sites.. J Mol Biol.

[16] Képès F (2004). Periodic transcriptional organization of the *E. coli* genome.. J Mol Biol.

[17] Wright MA, Kharchenko P, Church GM, Segre D (2007). Chromosomal periodicity of evolutionarily conserved gene pairs.. Proc Natl Acad Sci U S A.

[18] Cook PR (2002). Predicting three-dimensional genome structure from transcriptional activity.. Nat Genet.

[19] Marenduzzo D, Faro-Trindade I, Cook PR (2007). What are the molecular ties that maintain genomic loops?. Trends Genet.

[20] Bartlett J, Blagojevic J, Carter D, Eskiw C, Fromaget M (2006). Specialized transcription factories.. Biochem Soc Symp.

[21] Mastrangelo IA, Courey AJ, Wall JS, Jackson SP, Hough PV (1991). DNA looping and Sp1 multimer links: a mechanism for transcriptional synergism and enhancement.. Proc Natl Acad Sci U S A.

[22] Zeller RW, Griffith JD, Moore JG, Kirchhamer CV, Britten RJ (1995). A multimerizing transcription factor of sea urchin embryos capable of looping DNA.. Proc Natl Acad Sci U S A.

[23] Ptashne M, Gann A (1997). Transcriptional activation by recruitment.. Nature.

[24] Matthews KS (1992). DNA looping.. Microbiol Rev.

[25] Vilar JMG, Leibler S (2003). DNA looping and physical constraints on transcription regulation.. J Mol Biol.

[26] Zurla C, Manzo C, Dunlap D, Lewis D, Adhya S (2009). Direct demonstration and quantification of long-range DNA looping by the *λ* bacteriophage repressor.. Nucleic Acids Res.

[27] Sumedha, Weigt M (2008). A thermodynamic model for the agglomeration of DNA-looping proteins.. J Stat Mech.

[28] Strick TR, Dessinges MN, Charvin G, Dekker NK, Allemand JF (2003). Stretching of macromolecules and proteins.. Rep Prog Phys.

[29] Hagerman PJ (1988). Flexibility of DNA.. Annual review of biophysics and biophysical chemistry.

[30] Martin RG, Gillette WK, Martin NI, Rosner JL (2002). Complex formation between activator and RNA polymerase as the basis for transcriptional activation by MarA and SoxS in *Escherichia coli*.. Mol Microbiol.

[31] De Gennes PG (1988). Scaling concept in polymer physics.

[32] Grosberg AY, Khokhlov AR (1997). Statistical Physics of Macromolecules.

[33] Bloomfield VA (1997). DNA condensation by multivalent cations.. Biopolymers.

[34] Englander J, Klein E, Brumfeld V, Sharma AK, Doherty AJ (2004). DNA toroids: framework for DNA repair in *Deinococcus radiodurans* and in germinating bacterial spores.. J Bacteriol.

[35] Stukan MR, Ivanov VA, Grosberg AY, Paul W, Binder K (2003). Chain length dependence of the state diagram of a single stiff-chain macromolecule: Theory and monte carlo simulation.. J Chem Phys.

[36] Langowski J (2006). Polymer chain models of dna and chromatin.. Eur Phys J E.

[37] Jackson DA, Iborra FJ, Manders EM, Cook PR (1998). Numbers and organization of RNA polymerases, nascent transcripts, and transcription units in HeLa nuclei.. Mol Biol Cell.

[38] Vaquerizas JM, Kummerfeld SK, Teichmann SA, Luscombe NM (2009). A census of human transcription factors: function, expression and evolution.. Nat Rev Genet.

[39] Rosa A, Everaers R (2008). Structure and dynamics of interphase chromosomes.. PLoS Comput Biol.

[40] Nicodemi M, Prisco A (2009). Thermodynamic pathways to genome spatial organization in the cell nucleus.. Biophys J.

[41] Xu M, Cook PR (2008). Similar active genes cluster in specialized transcription factories.. J Cell Biol.

[42] Wagner A (1999). Genes regulated cooperatively by one or more transcription factors and their identification in whole eukaryotic genomes.. Bioinformatics.

[43] Cohen BA, Mitra RD, Hughes JD, Church GM (2000). A computational analysis of whole-genome expression data reveals chromosomal domains of gene expression.. Nat Genet.

[44] Kosak ST, Scalzo D, Alworth SV, Li F, Palmer S (2007). Coordinate gene regulation during hematopoiesis is related to genomic organization.. PLoS Biol.

[45] Dostie J, Richmond TA, Arnaout RA, Selzer RR, Lee WL (2006). Chromosome Conformation Capture Carbon Copy (5C): a massively parallel solution for mapping interactions between genomic elements.. Genome Res.

[46] Jhunjhunwala S, van Zelm MC, Peak MM, Cutchin S, Riblet R (2008). The 3D structure of the immunoglobulin heavy-chain locus: implications for long-range genomic interactions.. Cell.

