# Supplementary material for: Spatial and Topological Organization of DNA Chains Induced by Gene Co-localization
Source: PLoS Comput Biol. 2010 Feb 12;6(2):e1000678. doi: 10.1371/journal.pcbi.1000678 (PMC2820526; doi:10.1371/journal.pcbi.1000678)
Supplement: Text S1 — 4 sections 1. Physics of self-avoiding worm like chains 2. Framework and calculation strategy 3. Free energy calculations 4. Insights into the condensed phases (0.70 MB PDF) [file pcbi.1000678.s001.pdf]

Supplementary material: Spatial and topological organization of DNA chains induced by gene co-localization

Ivan Junier
Institut des Systèmes Complexes Paris Île-de-France
57-59 rue Lhomond, F-75005, Paris, France

Epigenomics Project, Genopole®, CNRS UPS 3201
UniverSud Paris, University of Evry
Genopole Campus 1 - Genavenir 6, 5 rue Henri Desbruères
F-91030 Evry cedex, France

Olivier C. Martin
Univ Paris-Sud, UMR 8626 LPTMS, F-91405, Orsay
Univ Paris-Sud, UMR 0320 / UMR 8120 Génétique Végétale,
F-91190, Gif/Yvette, France

François Képès
Epigenomics Project, Genopole®, CNRS UPS 3201
UniverSud Paris, University of Evry
Genopole Campus 1 - Genavenir 6, 5 rue Henri Desbruères
F-91030 Evry cedex, France

1 Physics of self-avoiding worm like chains

If we ignore the fine structure of our model coming from the existence of discrete binding sites, we can understand the *macroscopic* behavior of the polymer by making the self-attraction uniform. It is thus appropriate to recall here the state diagram of a self-avoiding worm-like chain (WLC) with self-attracting interactions. In the absence of such an interaction, the WLC behaves as a self-avoiding random walk, at least on length scales larger than the persistence length. This leads to a "swollen" state where the radius of gyration R of the chain grows as a power of its contour length L, $R \sim L^{\nu}$ with $\nu \approx 0.588$ being the self-avoiding walk exponent [3].

Now, increase the attractive self-interaction of the polymer; contacts will be established in a parsimonious fashion. This will not change the exponent ν, so the swollen state extends to some critical value of the interaction potential. Beyond that value, energy effects dominate over entropic ones and the self-attracting polymer goes to a more compact conformation with $\nu = 1/3$. The transition "swollen" to "compact" can be first or second order depending on the interaction parameters and the persistence length of the polymer [3].

To get insights into the conformations arising in the compact phase, consider simply minimizing the energy, neglecting entropic effects. Then the most compact conformations are favored; to obtain high densities, a regular "packing" of the chain is preferable. This can be obtained by having the DNA wind many times around a circle, forming in effect a kind of torus. Accordingly, this has been called the "toroidal" state [3]. Clearly this structure has a very low energy, but its entropy is also low. If entropy plays a role (the temperature is not extremely low), less ordered conformations will be favored. This might lead to a "statistical globule" in which the DNA forms a ball but otherwise seems rather random.

In summary, there are three macroscopic conformations of the self-attracting WLC: one dilute state (swollen) and two compact states (globule and toroidal). Which is the equilibrium state depends on the parameters, the two most important ones being the attractive force and the polymer stiffness [4].

Coming back to our system consisting of a single chain with sparse interacting sites, its peculiarity is that only *a few designated sites* of the chain are subject to the attraction; this means that a further organization of the chain on smaller length scales can arise, as we now explain in detail.

2 Framework and calculation strategy

To estimate the free energy of the different states, we focus only on the bulk contribution (large radius limit) and we do not keep track of the associated numerical factors (scaling analysis). In the case of a sparse distribution of interacting sites, the position of the sites may be important for the conformation of the condensed phase. To simplify our discussion, we consider sites that are regularly spaced by Δ along the DNA.

In the most general case, the free energy of formation $F(R)$ of an isotropic polymer state (globular or swollen) of radius R can be decomposed into four terms:

$$F(R) = F_a(R) + F_b(R) + F_{ev}(R) + F_s(R) \tag{1}$$

$F_{\rm a}$ is the energy due to the attractive potential between the interacting sites; F_b is the contribution from the bending energy; F_{ev} is the free energy cost due to the excluded volume effect; F_s is the entropic cost for the polymer to remain within a region of radius R. For a given organization (swollen, homogeneous or micro-structured globule) of both the interacting sites and the polymer, these terms can be more or less important, which leads to an optimal size R_* that minimizes the free energy. The strategy then consists in computing R_*, and hence $F^* = F(R_*)$, for the different states by solving the corresponding equations $\partial F(R)|_{R_*} = 0$ in the large R limit.

3 Free energy calculations

In this section, we provide the derivation of the free energies for the homogeneous globule, the micro-structured globule and the swollen state. For the sake of clarity, we first recall the principles of single chain polymer theory that are useful for the derivation (section 3.1 and 3.2). Next, at the beginning of the sections 3.3, 3.4 and 3.5, we quickly recall the main contributions to the free energy for the studied case.

3.1 Statistical description of the WLC

First of all, at large scales the statistical behavior of the worm like chain (WLC) is well approximated by the behavior of a freely jointed chain polymer whose monomers have a length equal to the so-called Kuhn length, which is equal to two times the persistence length l_p of the WLC. As a result, in the following we consider the chain to consist of monomers $2l_p$ long so that the number of monomers N is equal to $L/2l_p$ – notice that

the present N is different from the N used in the numerical simulations, which is equal to the number of cylinders used to model the WLC. Finally, because of their crucial role in the thermodynamic description of the globule phases, we call ϕ the concentration of monomers inside a volume of radius R containing the whole chain and $\phi_i(r)$ the coarse-grained concentration of the interacting sites.

3.2 The excluded volume term F_{ev}

Excluded volume effects come from the fact that the monomers are hard-core objects that cannot overlap with each other: this reduction in the number of possible conformations leads to an entropy reduction. Considering only pairwise interactions, the overall free energy cost per k_BT, $F_{\mathrm{rep|pair}}/k_BT$, of such excluded volumes is therefore equal to the mean number of pair-interactions between the monomers. To evaluate this number, it is useful to divide the system into cells whose volume v_{ex} corresponds to the statistical range of the hard-core interaction due to a single monomer ($v_{\mathrm{ex}} \sim a l_p^2$), which is the very notion of excluded volume[1]. Then the probability for any two monomers to be at the same time in a given such cell is equal to $(v_{\mathrm{ex}}/R^3)^2$. Since there are R^3/v_{ex} cells, the probability for any two monomers to collide in the globule is therefore $(R^3/v_{\mathrm{ex}}) \times (v_{\mathrm{ex}}/R^3)^2 = v_{\mathrm{ex}}/R^3$. Finally, there are $\sim N^2$ possibilities of choosing two monomers so that:

$$F_{\mathrm{rep|pair}}/k_BT \sim N^2 \frac{v_{\mathrm{ex}}}{R^3}.$$

Using $\phi = N/R^3$, this relation can be written as $F_{\mathrm{rep|pair}} \sim R^3 \phi^2 v_{\mathrm{ex}} k_B T$. More generally, one can show that the simultaneous interaction of n monomers ($n \ll N$) leads to a repulsion term of the form:

$$F_{\mathrm{rep}|n-\mathrm{uplet}} \sim (R/v_{\mathrm{ex}})^3 (\phi\ v_{\mathrm{ex}})^n k_B T/n! \tag{2}$$

This expansion in n is at the base of the Flory theory for treating polymer collapse transitions [2]. This theory is of mean-field type as it is based on the hypothesis that the probability of a collision between any two monomers is calculated by supposing that the spatial motion of these monomers is not correlated, which is equivalent to neglecting correlations along the polymer (see [1]).

[1] The parameter a reflects the anisotropy of the excluded volume. It is typically equal to some fraction of l_p. For the sake of lightness, we use $a \sim l_p$, and hence, $v_{\mathrm{ex}} \sim l_p^3$, which does not change the interpretation of our results.

Having only the 2-monomer repulsion leads to a discontinuous collapse transition of the self-attracting WLC. To prevent this undesired collapse, it is sufficient to include 3 , 4, or higher monomer repulsions. Technically, it is easier to analyze the lowest orders so that we only keep the 2-monomer and 3-monomer repulsion terms, *i.e.*, $F_{\text{rep|pair}}$ and $F_{\text{rep|3-uplet}}$. Hence, using $v_{\text{ex}} \sim l_p^3$ and $\phi \sim \frac{L}{l_p R^3}$, we get:

$$F_{ev}(R) \sim k_B T \left(\frac{l_p L^2}{R^3} + \frac{l_p^3 L^3}{R^6} \right). \tag{3}$$

Notice that in the homogeneous globular phase, $F_{\text{rep|pair}}$ alone would lead to a complete collapse of the homogeneous phase ($R_* \to 0$) as in this situation $F_{\text{rep|pair}}$ is smaller than the attractive contribution at any scale R.

3.3 The homogeneous globule

For the homogeneous globule, we shall see that the free energy is dominated by the attractive potential and the 2-monomer plus the 3-monomer repulsions. Both the bending and the entropic contributions are negligible.

The bending energy of the WLC is given by $F_b = K \int_0^L ds \left(\frac{\partial \vec{t}}{\partial s}\right)^2$ where $\partial \vec{t}/\partial s$ is the variation of the tangent vector along the curvilinear abscissa s of the polymer and K is the bending modulus. For a semi-flexible ideal chain (E_b is then the only energy), one has $K = l_p k_b T$. Then, if the bending energy is associated with curvature on the order of $1/R$, one gets $F_b(R) \sim k_B T L l_p / R^2$.

In the globule phase, one has $R_* \sim L^{1/3}$ since the polymer is compact. One can then check that the bending term can always be neglected with respect to the attractive term, and the same holds for the entropic cost $F_s(R_*)$. Indeed, this last quantity can be evaluated as the entropy cost for a freely jointed polymer chain of $L/2l_p$ $2l_p$ long monomers to have a gyration radius (*i.e.*, a spatial extension) of size R_*, which is given by $k_B T R_*^2 / L l_p$ [1]. Overall, the free energy of the homogeneous globule can be taken as:

$$F_{\text{hom}}(R) \sim F_a(R) + F_{ev}(R) \tag{4}$$

To compute $F_a(R)$, we first note that one interacting site can contribute to the attractive energy by an amount of at most $-\bar{n}V_0/2$, $\bar{n}$ being the maximal number of neighboring interacting sites, which depends on the interaction range d^* of the potential $V(r)$ and on the thickness r_0 of the

polymer (excluded volume effects). Suppose that this range is such that the number of interacting neighbors is large enough so that this maximum energy is in fact reached. Then, for a given homogeneous concentration $\phi_i(R)$, the energetic contribution of a given site of volume $\sim r_0^3$ reads $-\bar{n}V_0 r_0^3 \phi_i(R)/2$. Multiplying this single contribution by the number of interacting sites L/Δ, using $\phi_i(R) = L/\Delta R^3$ and using relation (3), we obtain:

$$F_a(R) \sim -\bar{n}V_0 \frac{r_0^3 L^2}{\Delta^2 R^3} \tag{5}$$

As a result:

$$\begin{aligned} F_{\text{hom}}(R) &\sim -\alpha\, k_B T \frac{L^2 l_p}{R^3} + \frac{L^3 l_p^3}{R^6} k_B T \\ \alpha &= -1 + \left(\frac{\Delta}{r_0}\right)^{-3} \frac{\bar{n}V_0}{k_B T}\,\frac{\Delta}{l_p} \end{aligned} \tag{6}$$

The value R_* that minimizes $F_{\text{hom}}(R)$ (obtained by the condition $\partial F_{\text{hom}}(R)|_{R_*} = 0$) reads:

$$\begin{aligned} R_* &= \left(\frac{2}{\alpha}\right)^{1/3} l_p \left(\frac{L}{l_p}\right)^{1/3} \quad \text{if } \alpha > 0 \\ R_* &\to \infty \quad \text{if } \alpha \leq 0 \end{aligned} \tag{7}$$

Therefore, a homogeneous globule is stable only if the parameters are such that $\alpha > 0$, *i.e.,* $V_0 > \frac{l_p \Delta^2}{r_0^3 \bar{n}} k_B T$. In this case:

$$F^*_{\text{hom}} \sim -\alpha^2 k_B T \frac{L}{l_p} \tag{8}$$

For $\alpha \leq 0$, we are driven away from the compact phase and then one cannot neglect anymore the entropic cost $F_s(R)$. This leads us to study now the case of the swollen state.

3.4 The swollen state

The swollen state is the result of a balance between the 2-monomer repulsion, the entropic cost and the attractive interaction.

When $\alpha \leq 0$, the equilibrium phase cannot be the compact globular phase. In this situation, the equilibrium state is the swollen state where the size of the polymer ball stems from the competition between the 2-monomer

repulsion term, the attractive and the entropic terms; the other terms (3-monomer repulsion and bending energy) can be neglected. Using the results derived in the previous section, one can write:

$$F_{\mathrm{swo}}(R) \sim \alpha\, k_B T \frac{L^2 l_p}{R^3} + k_B T \frac{R^2}{L l_p} \tag{9}$$

The value R_* that minimizes $F_{\mathrm{swo}}(R)$ ($\partial F_{\mathrm{swo}}(R)|_{R_*} = 0$) reads:

$$R_* = \left(\frac{3|\alpha|}{2}\right)^{1/5} l_p \left(\frac{L}{l_p}\right)^{3/5} \tag{10}$$

and

$$F^*_{\mathrm{swo}} \sim -|\alpha|^{2/5} k_B T \left(\frac{L}{l_p}\right)^{1/5} \tag{11}$$

When $|\alpha|$ is close to zero, one must include the bending energy, leading to the well-known ideal behavior of polymers at the so-called θ-temperature. One then finds $R_* \sim l_p(L/l_p)^{1/2}$ and $F^*_{\mathrm{ideal}} \sim k_B T (L/l_p)^{1/2}$.

3.5 The micro-structured globule

For the micro-structured globule, the calculation consists in first expressing the globule radius as a function of the distance between the foci. The latter is a relevant variable for computing the free energy. Next, we shall see that the free energy can be divided into two parts. One part concerns the interacting sites that are localized within the foci. In this respect, the calculation mainly consists in comparing the cost coming from the sites lying on the surface of the foci and those lying inside (surface tension problem). The other part concerns the free energy of the non-attracting part of the polymer that is located in between the foci. This part is dominated by the 2-monomer repulsion, plus the entropic cost and the bending contribution coming from the stretching of the polymer between the foci.

To treat the case of the micro-structured globule, we suppose i) that the interacting sites are located within foci of constant size r_f and ii) that two connected foci (*i.e.* by two consecutive interacting sites along the DNA) are separated by a distance x that is uniform across the whole globule. The first hypothesis is equivalent to saying that the foci are mostly free of non-interacting polymer so that the concentration $\phi_i(r_f)$ within a focus is close to its maximal value, *i.e.*, $\phi_i(r_f) \sim r_0^{-3}$, otherwise the concentration ϕ_i would decrease for larger foci. Thus, just as for $\bar{n}$ (see above), r_f is a function (that

we do not determine here) of the interaction range d^* and of the thickness of the polymer r_0. This hypothesis consists also in neglecting the variations from focus to focus in the value of ϕ_i, which can be seen as a mean-field approximation. The second hypothesis is also of mean-field nature since it consists in neglecting the spatial variations of the distance between the foci.

A direct consequence of hypothesis ii) is that the size R of the globule is a function of x. More precisely, in the bulk, a focus typically belongs to a single cell of volume $O(x^3)$. Since there are $(L/\Delta)/(r_f^3/r_0^3)$ foci, one has $R^3 \sim (L/\Delta) \times x^3/(r_f^3/r_0^3)$ so that:

$$R = R_x \sim \frac{r_0}{r_f} \left(\frac{L}{\Delta}\right)^{1/3} x \quad (12)$$

Now outside of the foci, the polymer is typically distributed in a random way so that the excluded-volume free energy is of the same nature as in relation (3) but with $R = R_x$. Also, in contrast with the homogeneous globule, the 2-monomer repulsion between two any monomers is not in competition with an attractive interaction because there are no binding sites outside of the foci. As a consequence, the stabilizing 3-monomer repulsion term can be dropped, leading to:

$$F_{ev}(x) \sim k_B T \frac{l_p L^2}{R_x^3} \sim k_B T \frac{r_f^3 l_p \Delta}{r_0^3 x^3} L \quad (13)$$

The bending energy of the polymer can be decomposed as the sum of the L/Δ bending contributions f_b coming from inter foci Δ-long chains. Supposing a curvature on the order of $1/x$ one gets $f_b \sim k_B T l_p \Delta / x^2$ so that:

$$F_b(x) \sim k_B T \frac{l_p}{x^2} L \quad (14)$$

The entropy cost can be divided into two parts. On the one hand, there is an overall cost S_{overall} that accounts for the constraint on the polymer to go from focus to focus. This can be seen as the entropy cost of a random walk of length L, whose unitary step is given by the typical distance x between the foci, and that has a spatial extension R_x. Hence, $S_{\text{overall}} \sim k_B T R_x^2 / L x \sim L^{-1/3}$, which vanishes at large L. On the other hand, every Δ-long inter-foci part of the polymer is stretched on the order of a distance x. Each one costs an entropy on the order of $k_B T x^2 / \Delta l_p$. Hence, L/Δ parts lead to a global contribution on the order of $k_B T x^2 L / \Delta^2 l_p$, which is much larger than S_{overall} at large L. As a result:

$$F_s(x) \sim k_B T \frac{x^2 L}{\Delta^2 l_p} \quad (15)$$

Finally, using the framework of [4] for evaluating the attractive free energy term, F_a can be seen as an overall contribution $-L\bar{n}V_0/\Delta$ that is compensated by surface penalties coming from each focus. For a given focus, the surface penalty is due to a smaller number, hereafter referred to as $\bar{n}_-$, of interacting neighbor sites at the surface. Within a focus, the number of surface sites is given by r_f^2/r_0^2 so that the surface penalty per focus reads $(\bar{n} - \bar{n}_-)V_0 r_f^2/r_0^2$. Multiplying by the number of foci $(L/\Delta)/(r_f^3/r_0^3)$, one gets $F_a \sim -V_0(\bar{n} - (\bar{n} - \bar{n}_-)r_0/r_f)(L/\Delta)$ so that:

$$F_a \sim -\bar{n}V_0 \frac{L}{\Delta} \tag{16}$$

This last approximation corresponds to the situation of gene co-localization where r_f is much larger than r_0 (see main text). Moreover, $\bar{n}_-$ is typically a finite fraction of $\bar{n}$. As a result:

$$F_{\text{het}}(x) \sim -\bar{n}V_0 \frac{L}{\Delta} + k_B T L \left(\frac{r_f^3 l_p \Delta}{r_0^3 x^3} + \frac{l_p}{x^2} + \frac{x^2}{\Delta^2 l_p} \right)$$

To further simplify the analytic treatment, we study the case of a not too small Δ/l_p. In this case, the bending term can be neglected with respect to the excluded volume term (see below) so that:

$$F_{\text{het}}(x) \sim -\bar{n}V_0 \frac{L}{\Delta} + k_B T L \left(\frac{r_f^3 l_p \Delta}{r_0^3 x^3} + \frac{x^2}{\Delta^2 l_p} \right) \tag{17}$$

The value x_* that minimizes this free energy $(\partial F_{\text{het}}(x)|_{x_*} = 0)$ reads:

$$x_* = \left(\frac{3}{2}\right)^{1/5} l_p \left(\frac{r_f}{r_0}\right)^{3/5} \left(\frac{\Delta}{l_p}\right)^{3/5}$$

At this point, one can justify dropping the bending term. To this end, we introduce the number of interacting sites per focus $n_I = (r_f/r_0)^3$. At $x = x_*$, the ratio of the bending energy l_p/x_*^2 to the repulsion term $r_f^3 l_p \Delta / r_0^3 x_*^3$ gives $n_I^{-12/5}(\Delta/l_p)^{-2/5}$, which is small even for a relatively modest number of interacting sites per focus. Coming back to the free energy calculation, one finds:

$$\begin{aligned} F^*_{\text{het}} &\sim -\alpha' \bar{n} V_0 \frac{L}{\Delta} \\ \alpha' &= 1 - n_I^{2/5} (\Delta/l_p)^{1/5} \frac{k_B T}{\bar{n} V_0} \end{aligned} \tag{18}$$

$$R_* = n_I^{-2/5} (\Delta/l_p)^{3/5} \left(\frac{L}{\Delta}\right)^{1/3} l_p \tag{19}$$

As a consequence, compared to the free energy of the swollen state and the homogeneous globule *the micro-structured globule is the equilibrium thermodynamic state if the parameters are such that* $\alpha' > 0$ *and* $\alpha'/\alpha^2 > \frac{k_B T}{\bar{n} V_0} \frac{\Delta}{l_p}$ as long as $\alpha > 0$; this last condition is satisfied for the spatial co-localization of genes.

4 Insights into the condensed phases

4.1 Order parameter

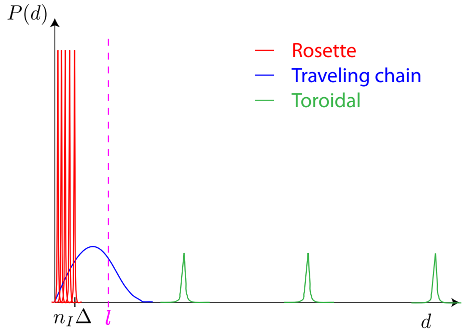

Our different topologies can be identified by defining an order parameter whose value reflects the topological structuring of the polymer. To distinguish between the solenoidal conformations, the rosette necklaces and the traveling chains, it is useful to characterize the distribution $P(d)$ of the distance d along the DNA that separates any two binding sites that are in contact in space, *i.e.* that belong to the same focus. Let's consider first a nearly ideal case where successive binding sites are at distances varying only slightly from Δ as measured along the DNA. For a pure necklace rosettes, $P(d) \approx n_I^{-1} \sum_{i=1}^{n_I} \delta(d - i \times \Delta)$ where $\delta(\bullet)$ is the Dirac (or Kronecker) distribution and n_I the mean number of interacting sites within a focus. For a perfect toroidal phase, $P(d)$ will have peaks at fixed (large) multiples of Δ, spread out to infinite d as the length of the chain diverges. In the traveling chain conformations, $P(d)$ will have contributions from d close to Δ, 2Δ, etc., whose height will not be sensitive to the length of the chain, but like the toroidal case, there will also be contributions for arbitrarily large d, corresponding to walks that come back after arbitrarily many steps. This situation is represented in the adjoining figure.

Mathematically, the rosette case corresponds to having the walk among foci be "transient": once one has gone away far enough, one never returns. The toroidal case has the property that the return probabilities are nearly periodic, spanning the whole range of d. Finally, the traveling chain case has a finite probability of returning in a finite number of steps *but* also has a finite probability of returning after $O(L)$ steps: it is thus intermediate between the two other cases.

Even when the inter-site distances fluctuate a lot around Δ, we can still

characterize $P(d)$ as above; furthermore, one can introduce an order parameter distinguishing the three topological structurings as follows. First define a distance l a few times greater than $n_I\Delta$; then consider the (topological) order parameter $t = \int_0^l dx\, P(x)$ as l grows but nevertheless is smaller than the period arising in the toroidal structure. This parameter satisfies:

$$t = 1 \quad \text{for a necklace of rosettes}$$
$$t \simeq 0 \quad \text{for a toroidal structure}$$
$$0 < t < 1 \quad \text{for a traveling chain conformation.}$$

4.2 Transition orders and the effects of disorder

The main difficulty for establishing the state diagram of our system lies in the high meta-stability of the solenoidal and traveling chain conformations. This can be illustrated for instance when one considers the frontier region between the swollen state and one of the condensed states, *e.g.* when the interacting sites are positioned periodically. To highlight the transition dynamics, we report single trajectories for the compaction into the traveling chain structure (or the rosette configuration if the polymer is small) in the following figure. Along these trajectories, we vary the amplitude of the attractive potential. The curves show the evolution of the internal energy $e = E/N$ per monomer of the system, N being here the number of cylinders used to model the polymer and is thus proportional to L. The strength V_0 of the potential is changed using different ramping velocities $v = |dV_0/dt|$. The energy per monomer e includes the polymer bending energy and the attraction energy between spatially close interacting sites. For the trajectory where the potential becomes less attractive (left to right arrow), the initial configuration is taken from the end of the compaction process with the lowest ramping velocities. Similarly, for the starting configuration when entering the compact phase, we start with a swollen configuration.

Our results strongly suggest a discontinuous transition, with strong hysteresis effects. On the time scales accessible to our computations, the swollen and condensed phases can coexist for intermediate values of the attractive potential (schematically indicated by the gray area). In effect, these states are metastable, and presumably are separated by high energy or entropy barriers. Moreover, as shown in the figure, the transition mechanism is not affected by adding a small amount of disorder in the position of the binding sites. In the figure, we have also indicated the typical conformation that is reached in the condensed phase. In this regard, from the last row, one can

see that in the condensed phase the small energy jumps correspond to the integration of isolated sites in already formed foci.

The parameters used for the figure are the following: naked DNA, $d^* = 6$ nm, and $\Delta = 4l_p$. The disorder consists in randomly shifting the positions of the binding sites around their periodic position according to a uniform distribution of amplitude $1.2 \times l_p$, an amount larger than $\Delta/4$.

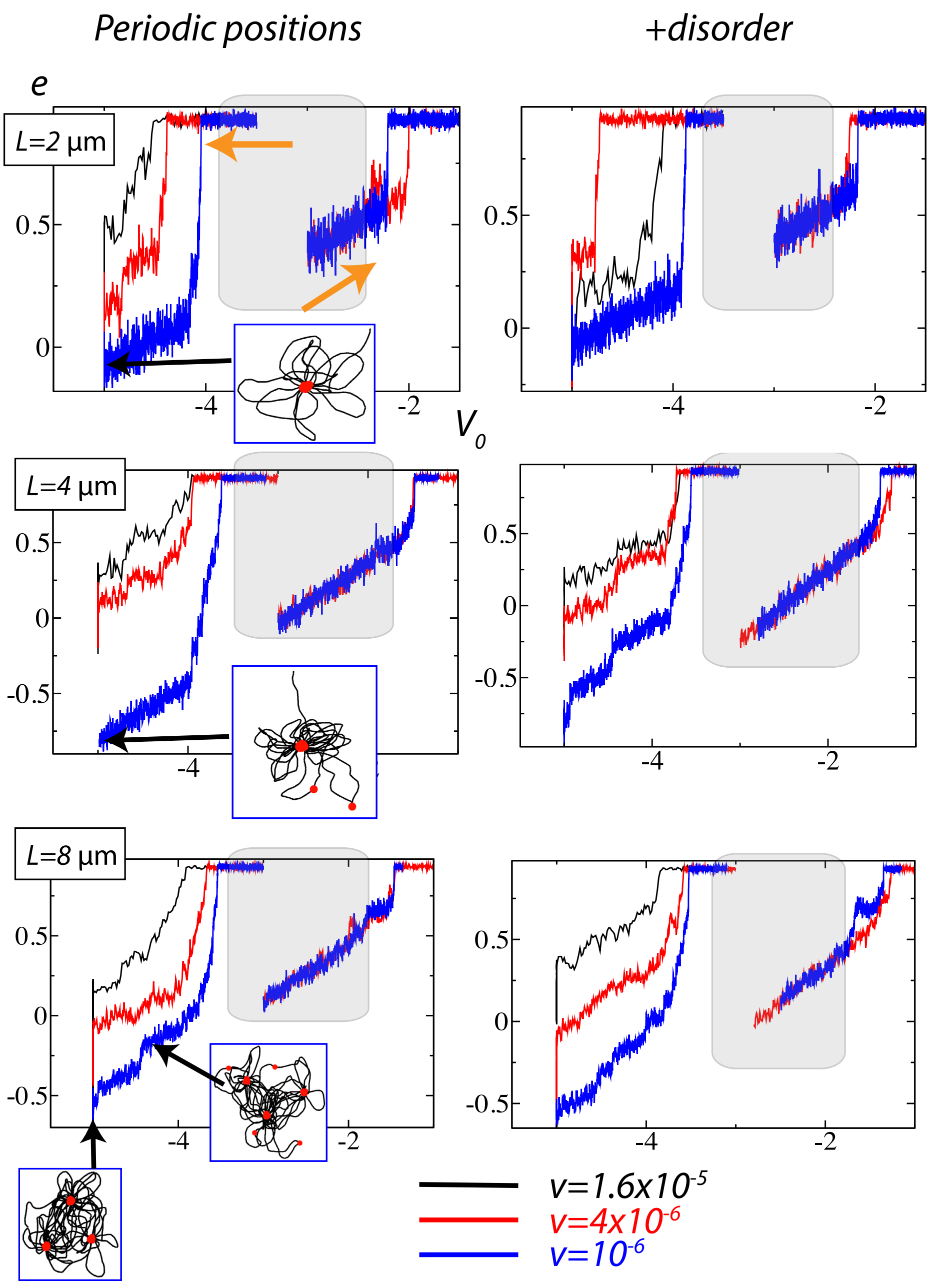

Periodic positions
+disorder
e
L=2 µm
L=4 µm
L=8 µm
V0
v=1.6x10-5
v=4x10-6
v=10-6

References

1. P.-G. De Gennes. *Scaling concept in polymer physics.* Cornell University Press, Ithaca, NY, 3rd edition, 1988.

2. P Flory. *Principles of Polymer Chemistry.* Cornell University Press, Ithaca, NY, 1971.

3. A. Y. Grosberg and A. R. Khokhlov. *Statistical Physics of Macromolecules.* AIP Press, 1997.

4. M. R. Stukan, V. A. Ivanov, A. Yu. Grosberg, W. Paul, and K. Binder. Chain length dependence of the state diagram of a single stiff-chain macromolecule: Theory and monte carlo simulation. *J Chem Phys*, 118(7):3392–3400, 2003.

Acknowledgments

This work was supported by the Sixth European Research Framework (project number 034952, GENNETEC project), PRES UniverSud Paris, CNRS and Genopole.
