# Supplementary material for: Spatial and Topological Organization of DNA Chains Induced by Gene Co-localization
Source: PLoS Comput Biol. 2010 Feb 12;6(2):e1000678. doi: 10.1371/journal.pcbi.1000678 (PMC2820526; doi:10.1371/journal.pcbi.1000678)
Supplement: Figure S1 — Discrete cylinder approximation of a self-avoiding worm-like chain model (0.09 MB PDF) [file pcbi.1000678.s002.pdf]

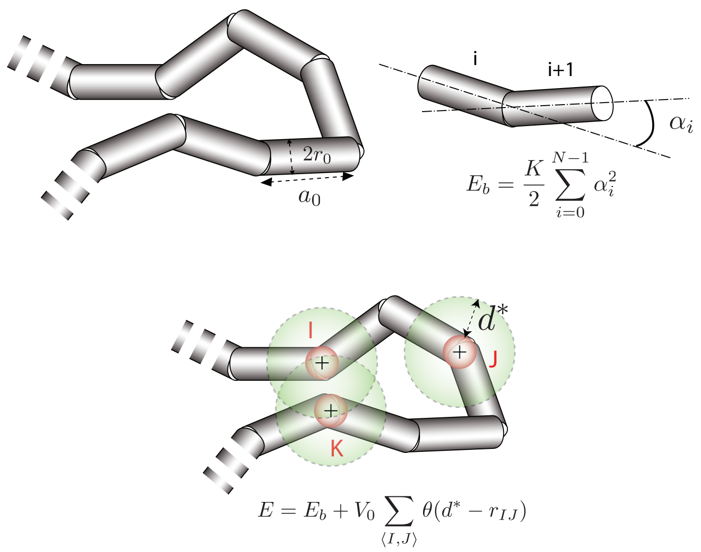

Figure 1: *Discrete cylinder approximation of a self-avoiding worm-like chain model.* Two upper panels: geometric parameters of the cylinders and the quantities specifying the bending energy E_b of a semi-flexible polymer. Lower panel: adding interactions between specific sites along the chain.
