# Supplementary material for: Spatial and Topological Organization of DNA Chains Induced by Gene Co-localization
Source: PLoS Comput Biol. 2010 Feb 12;6(2):e1000678. doi: 10.1371/journal.pcbi.1000678 (PMC2820526; doi:10.1371/journal.pcbi.1000678)
Supplement: Figure S2 — Rosette conformations (0.10 MB PDF) [file pcbi.1000678.s003.pdf]

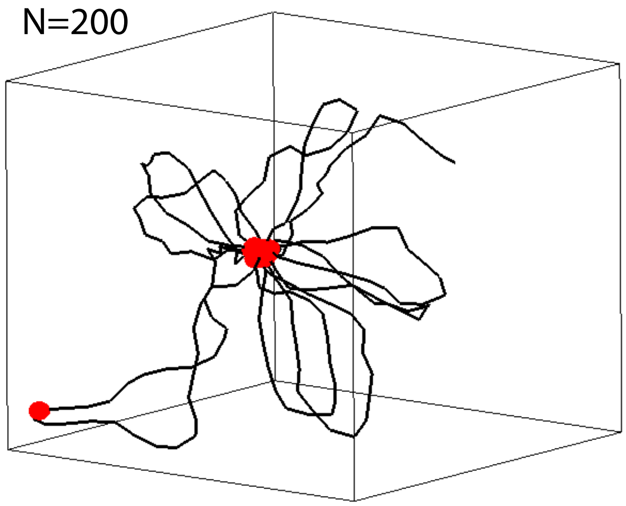

Figure 2: *Rosette conformations.* For few interacting sites along the polymer, the condensed phase of the polymer leads to a rosette like structure. Naked DNA parameters with $d^* = 6$ nm, $\Delta = 4l_p$ ($\Delta = 4l_p$) and $V_0 = 3.5\ k_BT$. $L = 2\ \mu$m.
