# Supplementary material for: Spatial and Topological Organization of DNA Chains Induced by Gene Co-localization
Source: PLoS Comput Biol. 2010 Feb 12;6(2):e1000678. doi: 10.1371/journal.pcbi.1000678 (PMC2820526; doi:10.1371/journal.pcbi.1000678)
Supplement: Figure S3 — Anisotropic structuring of the interacting sites (0.14 MB PDF) [file pcbi.1000678.s004.pdf]

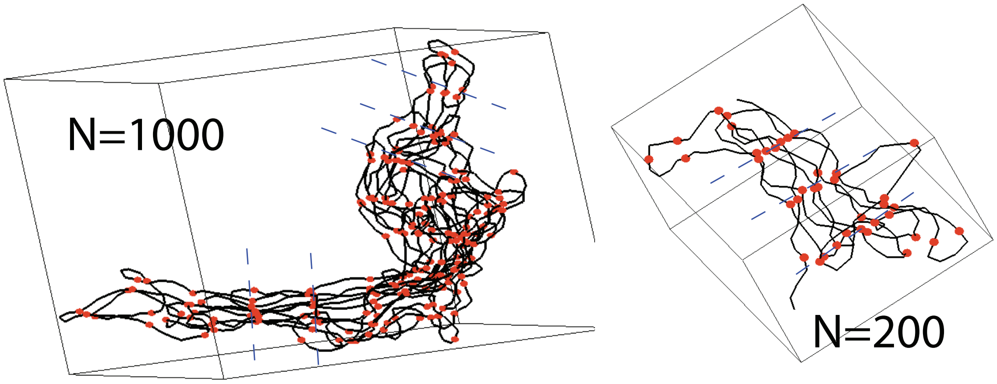

Figure 3: *Anisotropic structuring of the interacting sites.* In the case of chromatin fiber with periodic location of interacting sites, for $\Delta \sim l_p$, one finds peculiar chromosomal structuring where the interacting sites are organized along 1D lines or 2D planes as indicated by the blue dashes. This figure reports two snapshots of a typical conformation in the steady state for the $l_p = 150$ nm chromatin fiber with $\Delta = l_p$, $d^* = 10$ nm and $V_0 = 3.5\ k_BT$. Left panel: $L = 30\ \mu m$. Right panel: $L = 6\ \mu m$.
