# Supplementary material for: Spatial and Topological Organization of DNA Chains Induced by Gene Co-localization
Source: PLoS Comput Biol. 2010 Feb 12;6(2):e1000678. doi: 10.1371/journal.pcbi.1000678 (PMC2820526; doi:10.1371/journal.pcbi.1000678)
Supplement: Figure S4 — Choices for positioning the interacting site along the polymer (0.05 MB PDF) [file pcbi.1000678.s005.pdf]

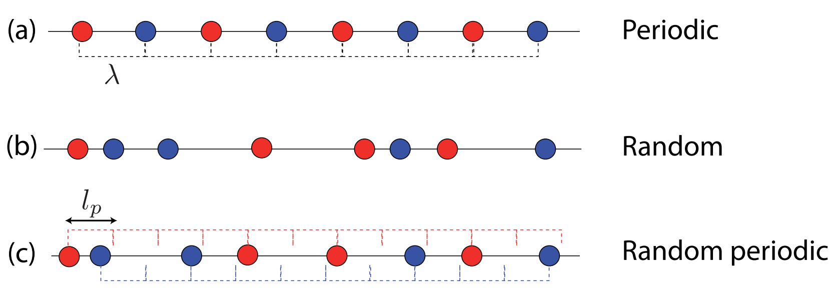

Figure 4: *Choices for positioning the interacting site along the polymer.* In our study, we investigate the folding properties for three classes of positionings, illustrated here with two types (red and blue) of binding sites. (a) The interacting sites are periodically positioned along the DNA and the type of the binding site is also periodic. (b) The sites are randomly positioned along the DNA, and the types are also random. (c) The sites are positioned along the DNA according to a random multiple of the persistence length l_p of the polymer, leading to partial order in position; however, the type of each binding site is chosen at random, so this case is referred to as “periodic random”.
