# Supplementary material for: Spatial and Topological Organization of DNA Chains Induced by Gene Co-localization
Source: PLoS Comput Biol. 2010 Feb 12;6(2):e1000678. doi: 10.1371/journal.pcbi.1000678 (PMC2820526; doi:10.1371/journal.pcbi.1000678)
Supplement: Figure S5 — Typical spatial conformation for a random positioning of the sites (0.12 MB PDF) [file pcbi.1000678.s006.pdf]

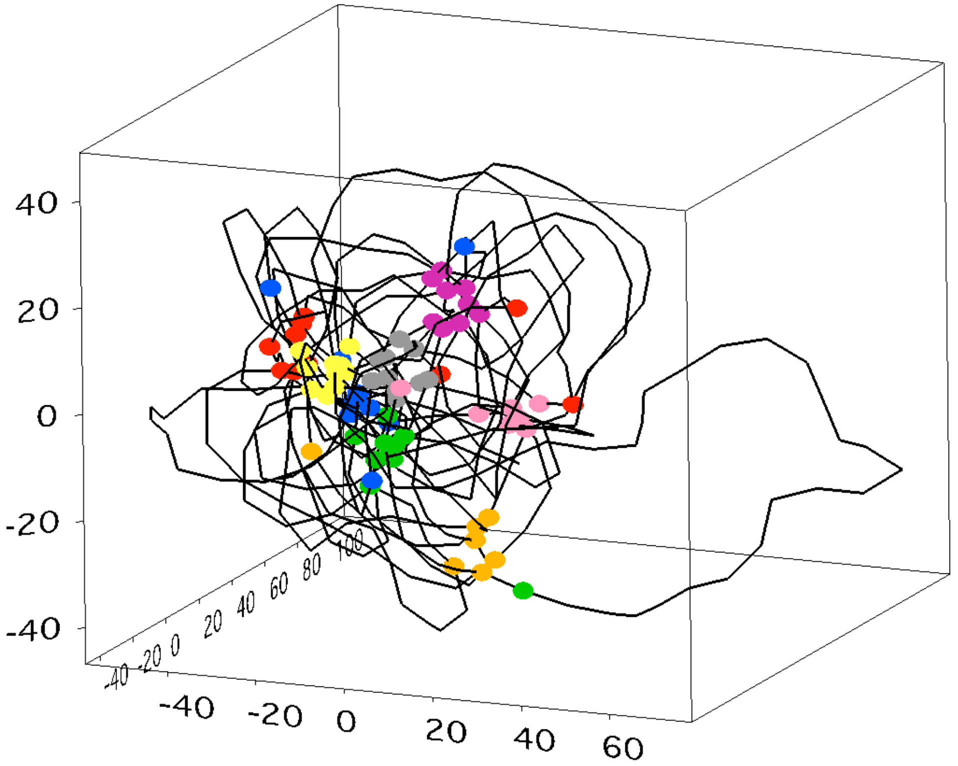

Figure 5: Typical spatial conformation for both the polymer and the interacting sites in a steady state for a random positioning of the sites. Note that several sites do not belong to any focus. Naked DNA, $N_t = 8$, $d^* = 6$ nm, $L = 8$ μm and $\lambda = 2l_p$.
